# Complexation of uranyl (UO_2_)^2+^ with bidentate ligands: XRD, spectroscopic, computational, and biological studies

**DOI:** 10.1371/journal.pone.0256186

**Published:** 2021-08-19

**Authors:** Abeer A. Sharfalddin, Abdul-Hamid Emwas, Mariusz Jaremko, Mostafa A. Hussien

**Affiliations:** 1 Department of Chemistry, Faculty of Science, King Abdulaziz University, Jeddah, Saudi Arabia; 2 King Abdullah University of Science and Technology (KAUST), Thuwal, Saudi Arabia; 3 Biological and Environmental Science and Engineering (BESE), King Abdullah University of Science and Technology (KAUST), Thuwal, Saudi Arabia; 4 Department of Chemistry, Faculty of Science, Port Said University, Port Said, Egypt; Nazarbayev University, KAZAKHSTAN

## Abstract

Three new uranyl complexes [(UO_2_)(OAc)_2_(CMZ)], [(UO_2_)(OAc)_2_(MP)] and [(UO_2_)(OAc)_2_(SCZ)] were synthesized and characterized by elemental analysis, FT-IR, UV-Vis spectroscopy, powder XRD analysis, and molar conductivity. The IR analysis confirmed binding to the metal ion by the sulfur and ethoxy oxygen atoms in the carbimazole (CMZ) ligand, while in the 6-mercaptopurine (MP) ligand, the sulfur and the N7 nitrogen atom of a purine coordinated binding to the metal ion. The third ligand showed a 1:1 molar ratio and bound via sulfonamide oxygen and the nitrogen of the pyrimidine ring. Analysis of the synthesized complexes also showed that acetate groups had monodentate binding to the (UO_2_^2+^). Density Functional Theory (DFT) calculations at the B3LYP level showed similar structures to the experimental results. Theoretical quantum parameters predicted the reactivity of the complexes in the order, [(UO_2_)(OAc)_2_(SCZ)] > [(UO_2_)(OAc)_2_(MP)]> [(UO_2_)(OAc)_2_(CMZ)]. DNA binding studies revealed that [(UO_2_)(OAc)_2_(SCZ)] and [(UO_2_)(OAc)_2_(CMZ)] have the highest binding constant (K_b_) among the uranyl complexes. Additionally, strong binding of the MP and CMZ metal complexes to human serum albumin (HSA) were observed by both absorbance and fluorescence approaches. The antibacterial activity of the complexes was also evaluated against four bacterial strains: two gram-negative; *Escherichia coli* and *Klebsiella pneumonia*, and two gram-positive; *Staphylococcus aureus* and *Streptococcus mutans*. [(UO_2_)(OAc)_2_(MP)] had the greatest antibacterial activity against *Klebsiella pneumonia*, the gram-positive bacteria, with even higher activity than the standard antibiotic. In vitro cytotoxicity tests were also performed against three human cancer lines, and revealed the most cytotoxic complexes to be [(UO_2_)(OAc)_2_(SCZ)], which showed moderate activity against a colon cancer cell line. Thus, uranyl addition enhances the antibacterial and anticancer properties of the free ligands.

## 1. Introduction

The majority of drugs are small organic molecules, which can be used as chelating agents that vary in the donor sites and influence biochemical processes by interacting with proteins, lipids, and other biomolecules. Enhancing their bioactivity by complexing them with metal ions has been used to design new and more effective medical drugs. Metal-based drug bioactivity can be increased by modulating the metal chelation, which in turn decreases their toxicity and enhances their lipophilicity, absorbance, and stability. The chemistry of actinides (*f*-block elements) coordination is rapidly growing in disease diagnostics due to their unique luminescent and magnetic properties [1]. These have been used in a wide range of clinical applications including diagnostics, photodynamic therapy, characterization of protein binding sites, and bioimaging [2]. Uranium is one of the rare earth deposit attracting attention from scientists due to its high chemical stability and low radiological risk [3, 4]. Among its applications in different fields, including the environmental field [5] and catalytic applications [6], uranyl complexes show biological activity as antimicrobial agents, which is particularly interesting. As one example, Ebrahimipour et al. had synthesized two Schiff base—UO_2_^2+^ complexes with only good efficiency against Gram-positive bacteria and mostly higher in comparison to its corresponding ligand [3, 7]. Another interesting uranyl complexes synthesized from Schiff base ligand have presented good antibacterial activity against Gram-positive and antifungal effects. These compounds were nanoscale and produced by the sonochemical method as a safe and eco-friendly approach [8]. Moreover, the complexation of the uranyl nitrate salt with mixed ligand also have enhanced the efficiency of the free ligands as antibacterial agents [9]. The binding affinity of (UO_2_^2+^) complexes with the most abundant biological molecules, DNA and HSA, also have been widely investigated as one of the double-edged sword of uranium. The luminescence feature of these complexes is used in their applications as spectroscopic probes for nucleic acids [10]. Thus, uranyl salts, nitrate, and acetate are commonly used as stains in electron microscopy as one of the biological applications. Generally, uranyl ions bind to proteins and lipids with sialic acid carboxyl groups, and to DNA and RNA by phosphate groups [11]. Therefore, the unique property of the uranyl ion as conformational changes for the structure of the biological molecule and a DNA cleavage estimated for biomedical and environmental applications [12, 13]

This study will investigate the interaction of three different drug molecules that have confirmed binding sites to uranyl (UO^2+^) and studied the effects of metal ion interactions on their biological properties. These drugs are approved and have been utilized as antithyroid (Carbimazole), anticancer (6-mercaptopurine) and antibiotic (Sulfaclozine) agents. The complexes obtained after reaction with uranyl acetate were characterized by various spectroscopic, fluorescence and thermal gravimetric methods to confirm the structures. DFT calculations were performed to provide insight into the coordination modules in the (UO_2_^2+^) complexes and their activities. There are different approaches to test the binding of the metal complexes to nucleic acid and protein molecules [14]. Among them, two methods, absorbance and fluorescence techniques, were used to evaluate the interaction mode and strength toward DNA or HSA molecules. The antibacterial activity of the synthesized uranyl complexes was evaluated against gram positive and negative microorganisms. Furthermore, cytotoxicity against three human cancer cell lines was also tested.

## 2. Experiments

### 2.1. Chemicals and synthesis procedure

Carbimazole (CMZ), 6-Mercaptopurine (6-MP), Sulfaclozine (SCZ), and uranyl acetate dihydrate were purchased from Sigma Aldrich. Also, the human serum albumin (HSA, A1887; globulin and fatty acid free) and the calf thymus DNA were purchased from Sigma Aldrich. The solvents were reagent grade and used as received, without further purification. The new complexes were synthesized as follows; 1mM of the metal salt was dissolved in ethanol and added dropwise to a freshly prepared solvent of CMZ (0.186g/25ml), 6-MP (0.152g/25ml), or SCZ (0.285 g/25 ml) with continuous stirring. The mixtures were run out in the refluxing system for 2-3h. The colored precipitates were collected by filtering and washed with cold ethanol. The complexes were kept in a desiccator over anhydrous CaCl_2_.

### 2.2. Characterization instruments

The microanalytical analyses (%carbon, %hydrogen and %nitrogen) were carried out in a Vario EL Fab. CHNS. The molar conductance of 10^−3^ M solutions for the complexes in DMF were measured on a HACH conductivity meter model. Infrared spectra for ligands and the complexes were recorded on a Bruker infrared spectrophotometer in the range of 400–4000 cm^−1^. The electronic spectra of the metal complexes carried out in Shimadzu UV/Vis spectrometer in the range of 200–800 nm. Fluorescence experiments were carried out on a Cary Eclipse spectrofluorometer from 300 to 600 nm. All the measurements were taken at room temperature for freshly prepared solutions. The PXRD data were collected on a Bruker D8 advance with a Cu Kα radiation wavelength of 0.15406 nm using a reported procedure ^11^. Thermogravimetric analysis TG-DTG experiments were conducted using a Mettler Toledo STARe thermal analysis system consisting of the STARe software. All experiments were performed using a single loose top-loading platinum sample pan under air at a flow rate of 30 mL/min and a 10°C/min heating rate for the temperature range 25–800°C. The content of the metal ions was calculated gravimetrically as metal oxides.

### 2.3. Theoretical calculation for the geometry and the binding affinity studies

The uranyl complexes [(UO_2_)(OAc)_2_(CMZ)], [(UO_2_)(OAc)_2_(MP)], and [(UO_2_)(OAc)_2_(SCZ)] were optimized using density functional theory (DFT) with the hybrid B3LYP exchange-correlation functional [15]. This level of theory with the SDD basis set has been verified for actinide and lanthanide complexes due to it producing reliable structural and energetic results [16]. The SDD basis set combines DZ with the Stuttgart-Dresden ECP basis set [17]. Di Bernardo et al., reported that this method generated computational results of reaction energies and vibrational frequencies of uranyl complexes in good agreement with experimental data [16]. The other elements, such as C, N, S, and O, were treated using the 6-31G(d,p) basis set. A vibration frequency calculation was performed to confirm that each structure was a minimum on the potential energy surface and without any imaginary frequencies. The obtained HOMO and LUMO energies and natural bond orbitals (NBO) were performed at the optimized geometries at the DFT / B3LYP level. The essential quantum parameters were calculated by the following equations; energy gap (E_gap_ = E_LUMO_ − E_HOMO_), absolute electronegativities (χ = −E_HOMO_ +E_LUMO_/2), absolute hardness (ɳ = E_LUMO_ − E_HOMO_/2), chemical potentials (μ = -χ), global softness (S = 1/2ɳ), and global electrophilicity (ω = π2/2ɳ) [7, 8]. Also, the MEP map was projected by applying the B3LYP/SDD level within DFT theory.

### 2.4. Estimation of the biological activity

#### 2.4.1. In vitro binding of the complexes to DNA

The DNA binding experiments were carried out by two standard techniques, absorption spectral traces and emission spectroscopy. Briefly, a titration experiment was performed between the constant concentration of the tested sample, the free ligand or the metal complexes, and a range (1.57–5.15 μM) of the buffer DNA solution. The obtained mixture was allowed to incubate for 5 min before recording the absorbance. The reference solution contained an equal amount of DNA volume that was added to the compound solution to eliminate the absorbance of the CT DNA itself, and Tris buffer was subtracted through baseline correction.

The binding constant K_b_ was computed with the Benesi-Hildebrand equation, which is defined as:

$$[\text{DNA}]/\left(\varepsilon_{\text{a}}-\varepsilon_{\text{f}}\right)=[\text{DNA}]/\left(\varepsilon_{\text{b}}-\varepsilon_{\text{f}}\right)+1/K_{\text{b}}\left(\varepsilon_{\text{a}}-\varepsilon_{\text{f}}\right).$$

where [DNA] = the concentration of CT-DNA in base pairs

ɛ_a_ = extinction coefficient observed for the A_obs_/[compound] at the given DNA concentration

ɛ_f_ = extinction coefficient of the free compound in solution

ɛ_b_ = extinction coefficient of the compound when binding to DNA.

The slope-to-intercept ratio was collected by plotting [DNA]/(*ε*_b_-*ε*_f_) versus [DNA], which is the K_b_ value.

The fluorescence properties of the metal complexes were utilized to study the effect of adding a varied concentration of DNA by Cary Eclipse spectrofluorometer. The fluorescence quenching constant (KSV) can be evaluated using the Stern–Volmer equation [18];

$$\text{Fo}/\text{F}\left(\text{or}\;\text{I}0/\text{I}\right)=1+\text{KSV}\;[\text{DNA}]$$

Where F_o_ or I_0_ is the fluorescence intensity in the absence of quencher while F or I is the fluorescence intensity in the presence of DNA. K_SV_ is the Stern–Volmer quenching constant. KSV can be obtained from the slope of the plot of F_o_/F_vs_. [DNA] or the concentration of the tested drug [18, 19].

#### 2.4.2. In vitro binding of the complexes to HSA

The concentration of HSA was determined from the absorption spectra taken at 280 nm and extinction coefficient 35,219 M^-1^ cm^-1^ with quartz cuvettes of 1 cm path-length. The method and the binding constant calculations were described earlier [20].

For the fluorescence technique, the excitation of HSA and emission wavelength were set at 280 and 300–400 nm, respectively. The interaction of HSA solution content of fixed concentration was tested with increasing concentrations of the complex starting at 0.07 to 0.25 M. The fluorescence constant (K_SV_) can be evaluated using the Stern–Volmer equation [21]; F_o_/F (or I_0_/I) = 1 + K_SV_ [DNA], where F_o_ is the fluorescence intensity in the absence of quencher, while F is the fluorescence intensity in the presence of uranyl complexes. K_SV_ is the Stern–Volmer quenching constant. The value of K_SV_ can be obtained from the slope of the plot of F_o_/F_vs_. [Complex] [14].

#### 2.4.3. Antibacterial activity assays

An agar well diffusion method was used to test the antibacterial activity of the synthesized complexes against four bacteria types: *Staphylococcus aureus* and *Streptococcus mutans* (Gram-positive bacteria), *Escherichia coli*, *Klebsiella pneumonia* (Gram-negative bacteria) using nutrient agar medium. All the bacteria was Ampicillin, the standard drug for Gram-positive bacteria, was used as a reference, while Gentamicin was used for the Gram-negative bacteria. Initially, sterilized Petri dishes (20–25 ml, each petri dish) were filled with sterilized media and allowed to solidify at room temperature. Sterilized saline equivalent to McFarland 0.5 standard solution (1.5x 10^5^ CFU mL^-1^) was used to prepare the microbial suspension. The turbidity of the obtained solution was adjusted to OD = 0.13 using a spectrophotometer at 625 nm. A sterile cotton swab was dipped into the adjusted suspension and was flooded on the dried agar surface then allowed to dry for 15 minutes with the lid in place. The volume of the tested compound solution (100 μL) was added to wells of 6 mm diameter made in the solidified media. The plates were incubated at 37°C for 24 hrs. This experiment was carried out in triplicate and zones of inhibition were measured on the mm scale.

#### 2.4.4. Cytotoxicity assays

The antitumor activity of the synthesized compounds was determined using a colorimetric technique. Three human cancer cell lines, colon (CaCo-2), myeloma (SK-MM-1), and breast cancer (MCF-7) cells were obtained from the VACSERA Tissue Culture Collection Unit. The cells were propagated in Dulbecco’s modified Eagle’s medium (DMEM) supplemented with 10% heat-inactivated fetal bovine serum, 1% L-glutamine, HEPES buffer and 50μg/ml gentamycin. All cells were maintained at 37°C in a humidified atmosphere with 5% CO2 and were subcultured two times a week. The cytotoxicity of the samples was determined with the MTT protocol for each cancer cell line. The assay followed the formation of a monolayer sheet after incubation in 96-well tissue culture plates at 37°C for 24 hours [22, 23]. The tested sample stock solution was diluted with maintenance medium (RPMI, 2% serum) to produce different concentrations and tested in the wells, leaving three wells with maintenance medium only as controls. After incubating the plate for 24 hours at 37°C, MTT solution (5 μM tetrazolium salt in PBS) was added to each well and incubated for 1–5 hours until MTT metabolism was complete. After that, the media was discarded, and the plate was subjected to air drying to remove residue at room temperature. Two hundred microliters of DMSO was added to the plates to resuspend the formazan, and thoroughly mixed for 5 minutes on a shaking table. The optical density (OD) of each well was measured spectrophotometrically at 560 nm, and the background at 620 nm was subtracted. The results were directly associated with viable cell quantity. Each experiment was carried out three times to obtain standard deviation values.

## 3. Results and discussion

### 3.1 Elemental analysis and molar conductance measurements

The physical properties of the (UO_2_^2+^) complexes are listed in Table 1. The metal complexes showed a 1:1 molar ratio (M:L). The elemental analysis results were in good agreement with the suggested formula. Those compounds are stable in air and soluble only in DMF or DMSO solvents. The molar conductance values for the metal complexes were low (5.46 and 3.13 Ω^-1^ cm^2^ mol^-1^) and revealed non-electrolytic complexes. Both [(UO_2_)(OAc)_2_(SCZ)] and [(UO_2_)(OAc)_2_(CMZ)] decompose above 150°C, while [(UO_2_)(OAc)_2_(MP)] has a higher melting point.

**Table 1:** Physical and analytical data of the free ligand and the uranyl complexes.

Metal complex	M.Wt.	color	Λm Ω^-1^ cm^2^ mol^-1^	Melting point
CMZ	185.23	white	1	124
[(UO_2_)(OAc)_2_(CMZ)].H_2_O	574	dark sandy	3.13	240
MP	152.18	yellow	1	300
[(UO_2_)(OAc)_2_(MP)]	540	brick	3.13	310
SCZ	250.05	white	1.3	130
[(UO_2_)(OAc)_2_(SCZ)]	672	beige	5.46	169

### 3.2. Infrared spectroscopy (IR)

**Fig 1:**
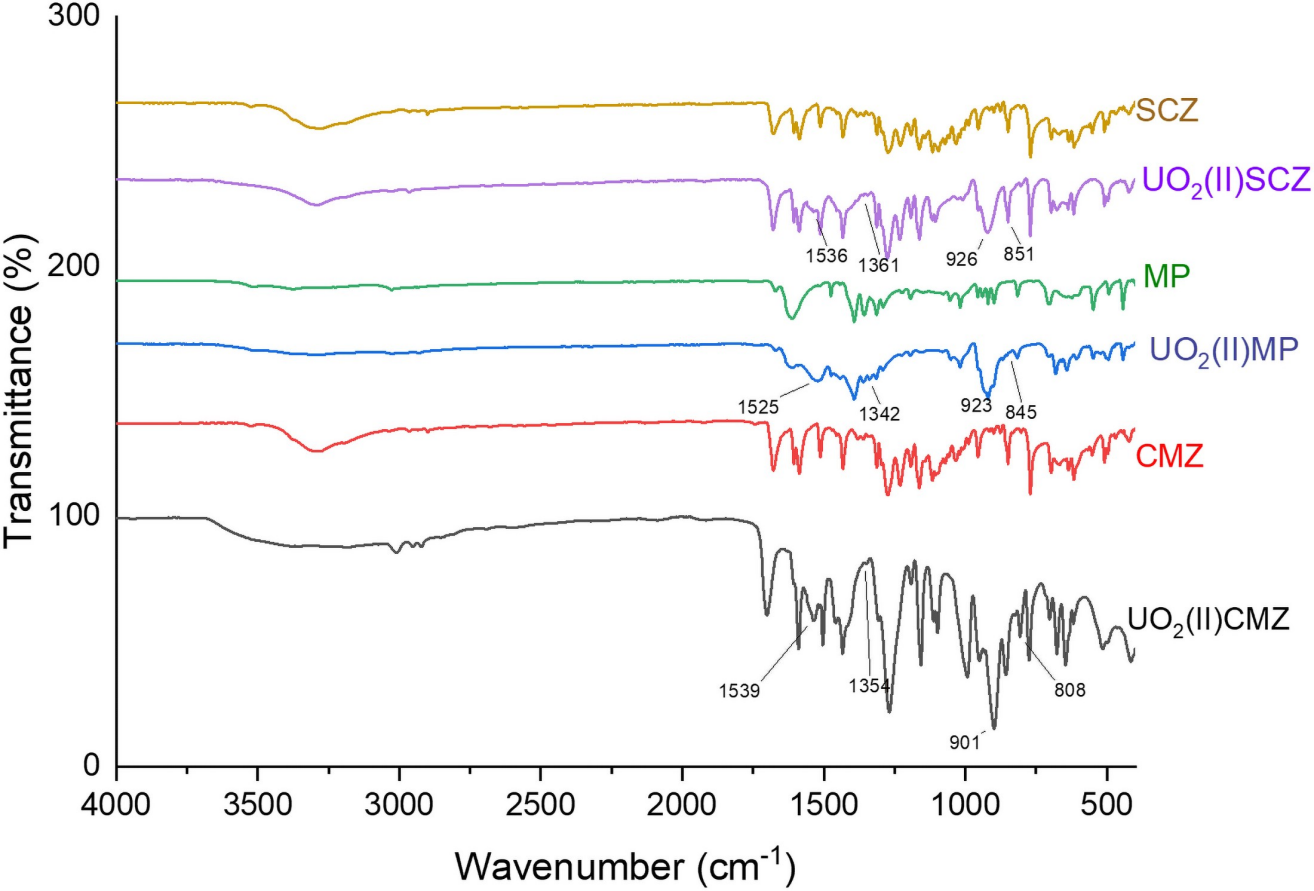
IR spectra of the free ligands (CMZ, MP and SCZ) compared to their (UO_2_^2+^) complexes.

Infrared spectroscopy is one approach used widely to investigate uranyl complexes. A comparison study between the FT-IR spectra of the free ligands (CMZ, MP, SCZ) and their uranayl complexes is presented in Fig 1. Moreover, the calculated vibrational frequencies using B3LYP/SDD were extracted to provide insight into the coordination mode(s) and binding properties.

Firstly, the CMZ spectrum showed three essential peaks corresponding to the sulfur atom, carbonyl oxygen, or ester oxygen. The ester oxygen group has two peaks, 1232 cm^-1^ for C-O-CH_3_ that disappeared after coordination to the metal ion, and 1163 cm^-1^ assigned to O-C_2_H_6_, which showed a blue shift to 1156 cm^-1^ compared to the free ligand spectrum. The sulfur stretching located between 1200–1193 [24] in the ligand displays a decrease in intensity that could be considered a strong indication that the sulfur atom is one of the donors in the metal complexes, as documented in the literature [25, 26].

The 6-MP ligand is known to bind to the metal by the sulfur and nitrogen of pyrimidine [26, 27]. The sharp peak at 1619 cm^-1^ for stretching C = N in the free ligand shifted to low frequency due to the deprotonation of the pyrimidine and imidazole rings. Another observation for the metal coordination was that the C = S stretching vibration at 1197 cm^-1^ presented a slight shift and reduction in the peak intensity [25, 26].

The SCZ free ligand has six donor sites: two pyrazine nitrogens, sulfonyl oxygens, sulfonamide nitrogen, and/or an amino group. The NH_2_ frequencies between 3291 and 2962 cm^-1^ are maintained in the metal complex, which revealed that it is not coordinated to the metal ion. There is a significant red shift for the asymmetric band for the SO_2_ group, while the symmetric band disappeared upon the binding of one sulfonyl oxygen to the (UO_2_^2+^). Stretching vibration bands of aromatic (C = N) bands that appeared at 1682–1587 cm^-1^ in the SCZ spectrum shifted to low frequency after complexing.

The presence of the acetate groups in the uranyl complexes were confirmed by the new non-ligand bands observed in the range of 900 and 800 cm^-1^, and characterized the asymmetric and symmetric vibration of the uranyl entity indicating that they are nearly linear [7].

To estimate the U = O bond length in the complex, the force constant Fu-o (mdyn A^- 1^) was calculated using McGlynn ’s Equation [28]:

$${\left(\text{v}3\right)}^{2}={\left(1307\right)}^{2}*\text{Fu-o}/14.103.$$

The force constant was obtained, then substituted into the relation given by Jones [29]

$$\text{ru-o}=1.08{\left(\text{Fu-o}\right)}^{-1/3}+1.17,$$

The data are given in Table 2. The calculated Fu-o and ru-o values are fitted with the range of 7.018 mdyn A° and 1.734–1.779A°, respectively, found from various uranyl complexes [28]. Moreover, the υ_asy_(OCO) and υ_s_(OCO) of the acetate group in the uranyl complexes were used to study its binding behavior to the metal ion, which is located at 1540–1525 cm^-1^ and 1365–1345 cm^-1^, respectively, in metal complexes.

**Table 2:** Vibrational frequencies for the indicated band in the metal complex and the calculated ones.

Metal complex	[(UO_2_)(OAc)_2_(CMZ)]		[(UO_2_)(OAc)_2_(MP)]		[(UO_2_)(OAc)_2_(SCZ)]	
	EXP.	DFT	EXP.	DFT	EXP.	DFT
υ(UO_2_)_sym_	807	839	836	842	849	940
υ(UO_2_)_asym_	900	919	946	932	925	921
r_U-O_	1.732	1.799	1.734	1.796	1.736	1.792
υ (COO)_asym_	1540	1570	1536	1600	1538	1568
υ (COO)_sym_	1354	1297	1342	1352	1345	1291
ΔV = (V_*5*_*-*V_*4*_*)*	186	273	182	327	178	277
M-N	-	-	524	501	677	687
M-O	500	520	-	-	423	503
M-S	676	710	682	667	-	-

In general, if the calculated value of the free urynal salt Δ (OCO) = υ_asy_(OCO) − υ_s_(OCO), is greater than the Δ’ (OCO) = υ_asy_(OCO) − υ_s_(OCO) for the metal complex, this reveals the monodentate character of the acetate group. Vice versa, a smaller value reveals the bidentate character [30]. The calculated Δ’ (OCO) = υ_asy_(OCO) − υ_s_(OCO) for the prepared complexes were in the range of the monodentate character [30].

The formed complexes were confirmed by the appearance of two new nonligand bands between 680 cm^-1^, 670 and around 430 cm^-1^ due to M-S, M-N and M-O, respectively.

The experiment results can be compared to the calculated data presented in Table 2. The formed mono uranyl acetate complex theoretical frequency values are overestimated by 10–25 cm^-1^ as compared to the experimental values. This is because the DFT calculation was in the harmonic oscillator approximation, and without addition of the solvent effects [15].

### 3.3. Coordination geometry

**Fig 2:**
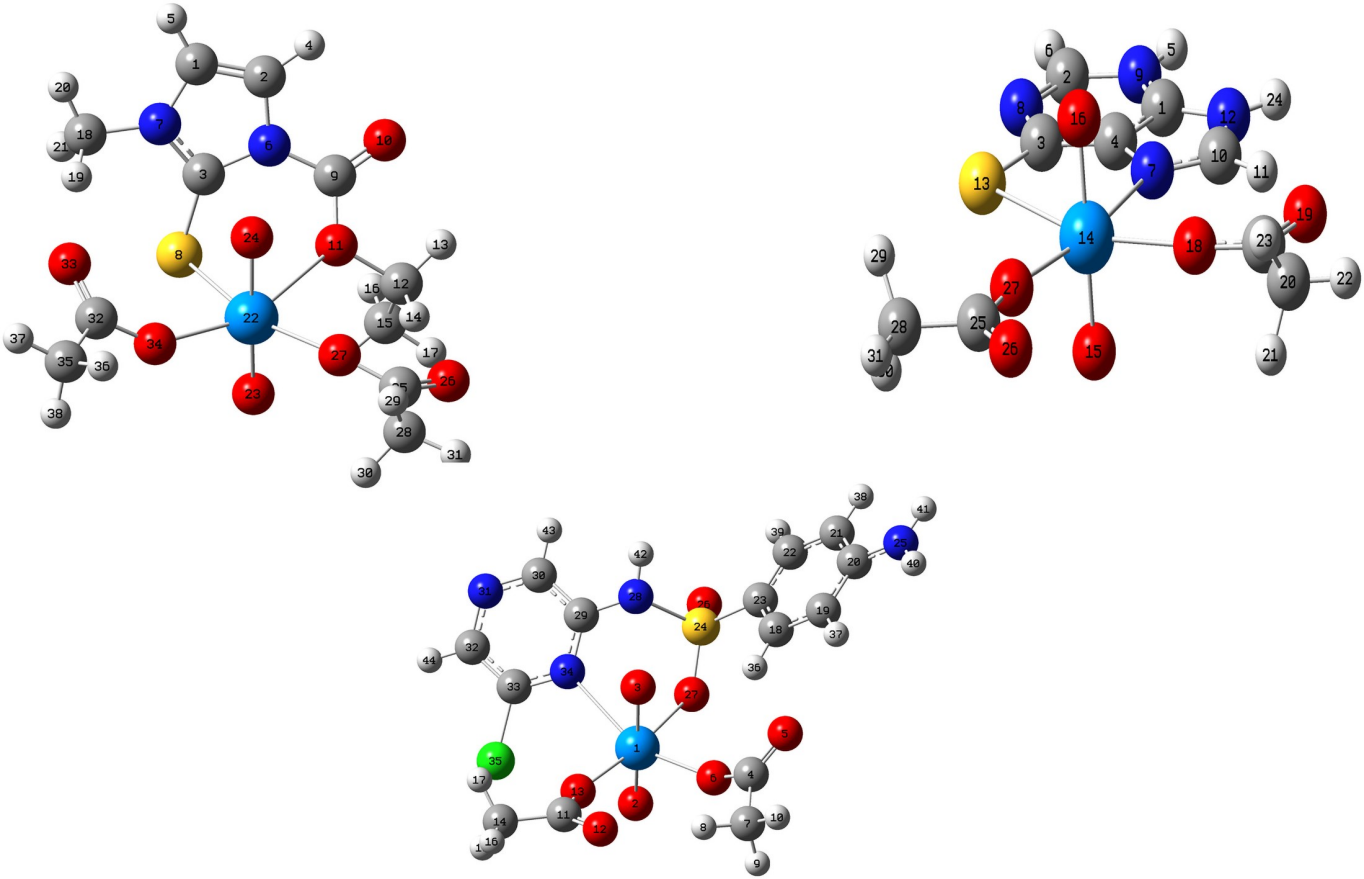
The optimized geometry and numbering system for uranayl metal complexes.

The obtained data from the IR, the elemental, and the conductivity analyses were used to build the input files for the DFT calculation. The optimized gas-phase geometries of the investigated (UO_2_)^2+^ complexes are shown Tables 3–5, along with the bond distances and the numbering system of the structures in the gas phase, Fig 2. Theoretical values of free ligands from our previous studies were used to investigate the change around the metal ion after the complexation. The bond between the O11 atom and C9 in the CMZ ligand was shorter than the free ligand by 0.06 Å, while the bond of S8-C3 was maintained after the reaction. The calculated value of the bond length of U-O in the free metal acetate, 1.769 Å [15], elongated to 1.799 Å due to the binding to the bidentate ligand, consistent with additional ligand-to-metal charge transfer. Moreover, the bond of U-O with the acetate molecule was reduced from the experimental value of 2.394 Å [15] to 2.18 Å. The bond angle S8-C3-N6 in the CMZ ligand became smaller by 2.79° due to the binding of the S atom to the (UO_2_^2+^). In contrast, the bond angle of C9-O11-C12 became bigger after complexation with the metal ion.

**Table 3:** Geometric (Å, deg) comparison of free ligand/ uranyl acetate and calculated structures for [(UO_2_)(OAc)_2_(CMZ)].

Bond length			Bond angle		
	complex	Free		complex	Free
S8-C3	1.75	1.75	S8-C3-N6	128.71	131.5
O11-C9	1.37	1.43	C9-O11-C12	116.95	111.2
U = O	1.794	1.769	O = U = O	179	-
U-O_acetate_	2.18	2.39	O34-U-O24	89	-
U-S	2.89	-	O23-U-O27	90	-
U-O	2.69	-	S8-U-O11	69	-

**Table 4:** Geometric (Å, deg) comparison of free ligand/ uranyl acetate and calculated structures for [(UO_2_)(OAc)_2_(MP)].

Bond length			Bond angle		
	complex	Free MP		complex	Free
S13-C3	1.71	1.70	S13-C3-N8	121.94	121.5
N7-C4	1.39	1.40	C10-N7-C4	107.25	110.2
U = O	1.796	1.769	O = U = O	178	-
U-O _acetate_	2.15	2.39	O18-U-O15	91	-
U-S	3.12	-	O16-U-O27	92	-
U-N	2.57	-	S13-U-N7	69.3	-

**Table 5:** Geometric (Å, deg) comparison of free ligand/ uranyl acetate and calculated structures for [(UO_2_)(OAc)_2_(SCZ)].

Bond length			Bond angle		
	complex	Free		complex	Free
S24-O27	1.62	1.70	O27-S24-N28	101.48	121.5
N34-C29	1.37	1.40	C29-N7-C33	117.25	110.2
U = O	1.794	1.769	O = U = O	178	-
U-O _acetate_	2.16	2.39	O2-U-O13	93.5	-
U-O	2.44	-	O3-U-O27	90	-
U-N	2.77	-	O27-U-N34	69.6	-

The (UO_2_^2+^) complex with MP showed a small change in the bond length around the coordination atom sites. Additionally, the elongation of the U = O is smaller than the CMZ complex by 0.003 Å. This observation could be due to the ability of oxygen to donate its electrons as a charge transfer more than nitrogen atoms [15]. The change of the angle C10-N7-C4 in the complex indicates binding to the metal ion, while the change of S13-C3-N8 was negligible.

The [(UO_2_)(OAc)_2_(SCZ)] bond lengths, S24-O27 and N34-C29, extend after binding to (UO_2_^2+^). The angle bond O27-S24-N28 in the [(UO_2_)(OAc)_2_(SCZ)] is smaller than SCZ, whereas the C29-N7-C33 became smaller to reduce the strain of the formed hexagonal ring. The bond of U-O with the acetate molecule decreased to 2.16 Å.

The new bonds of the U-S, U-N, and U-O, displayed elongation upon complexation, and were longer than the typical ionic bonds, indicating that they have a covalent character [31]. The O = U = O angle has a liner character with a bond angle of 179°. The angle of the bond around the metal shows a distorted octahedral structure.

### 3.4 Determination of the reactivity parameters

**Fig 3:**
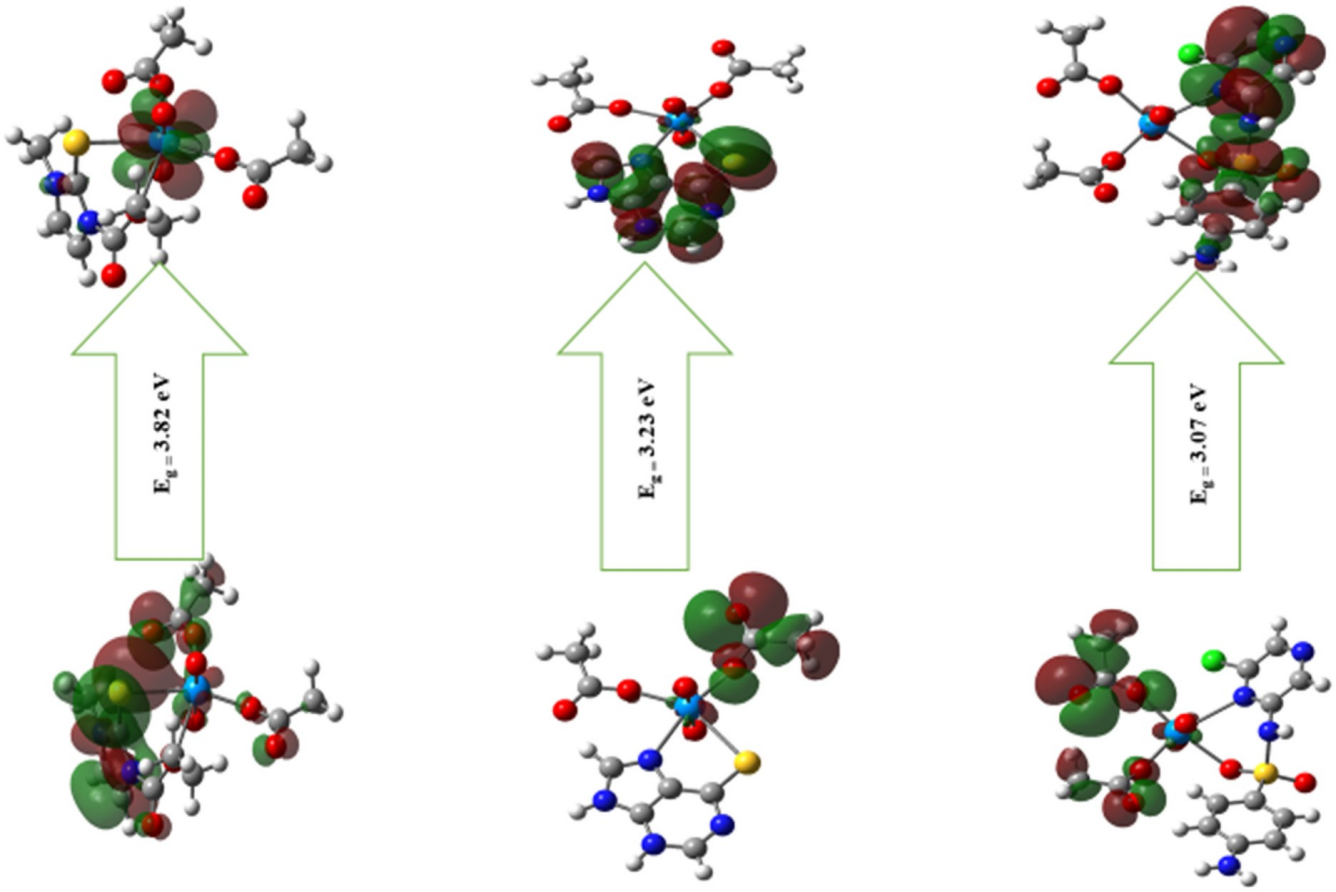
HOMO and LUMO orbitals of the investigated complexes using the B3LYP/SDD method.

The energies of the levels of the HOMO and LUMO, and the energy gap for the free ligand that was used in this study and the metal complexes were extracted to investigate the reactivity, stability, and chemical hardness and softness of these complexes. Energy levels of the HOMO, LUMO, and the energy gap for (UO_2_^2+^) bidentate complexes are shown in Fig 3. The HOMO orbital was distributed over the ligand molecule [(UO_2_)(OAc)_2_(CMZ)] while the LUMO was located in the uranyl atom orbital. In contrast, the HOMO of the [(UO_2_)(OAc)_2_(MP)] and UO_2_-SCZ were at the acetate molecules, and the LUMO orbitals were over the bidentate ligands.

From S1 Table, the quantum chemical descriptors, the HOMO orbital values of the uranyl complexes were higher than the free ligand assigned to the donor ability interaction. The reduction of the E_gap_ values after complexation with the metal ion was assumed to reflect the chemical reactivity and low stability of the tested complexes. The stability and reactivity of the molecules was of the order: [(UO_2_)(OAc)_2_(SCZ)] > [(UO_2_)(OAc)_2_(MP)] > [(UO_2_)(OAc)_2_(CMZ)], which indicates that [(UO_2_)(OAc)_2_(SCZ)] was the most reactive complex. The biological activity could be expressed by the electrophilicity index, which is the ability of an electrophile to gain an additional electronic charge from the amino base in the biological environment [32]. According to the electrophilicity values, [(UO_2_)(OAc)_2_(SCZ)] was considered to have potential biological activity.

### 3.5 Molecular electrostatic potential (MEPs) maps

**Fig 4:**
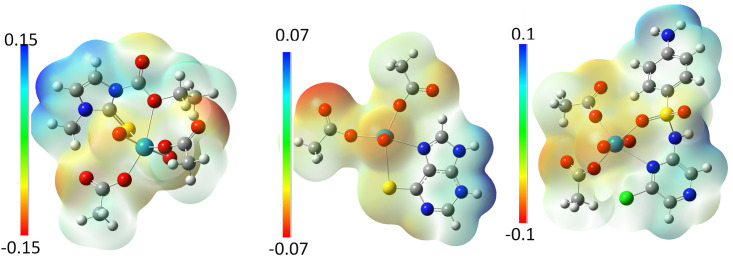
MEP maps of the UO_2_^2+^ -complexes calculated by the B3LYP/SDD method.

To locate the positive and negative charged electrostatic potential in the molecule, molecular electrostatic potential maps were used to predict the reactive sites in the tested complexes [33]. The calculated MEPs of the uranyl complexes were investigated using B3LYP/SDD, and are presented in Fig 4. The red color indicates the negative extreme zone (the minimum electrostatic potential), and the blue color indicates the positive extreme zone (the maximum electrostatic potential). The results showed that carbonyl groups in the acetate and the uranyl or in the ligand are electron-rich zones on the map, denoting sites. Most of the ligand molecules presented electrophilic spots and are assumed to be biological attackers of the phosphate backbone in the DNA or amino residue.

### 3.6 X-ray diffraction analysis

**Fig 5:**
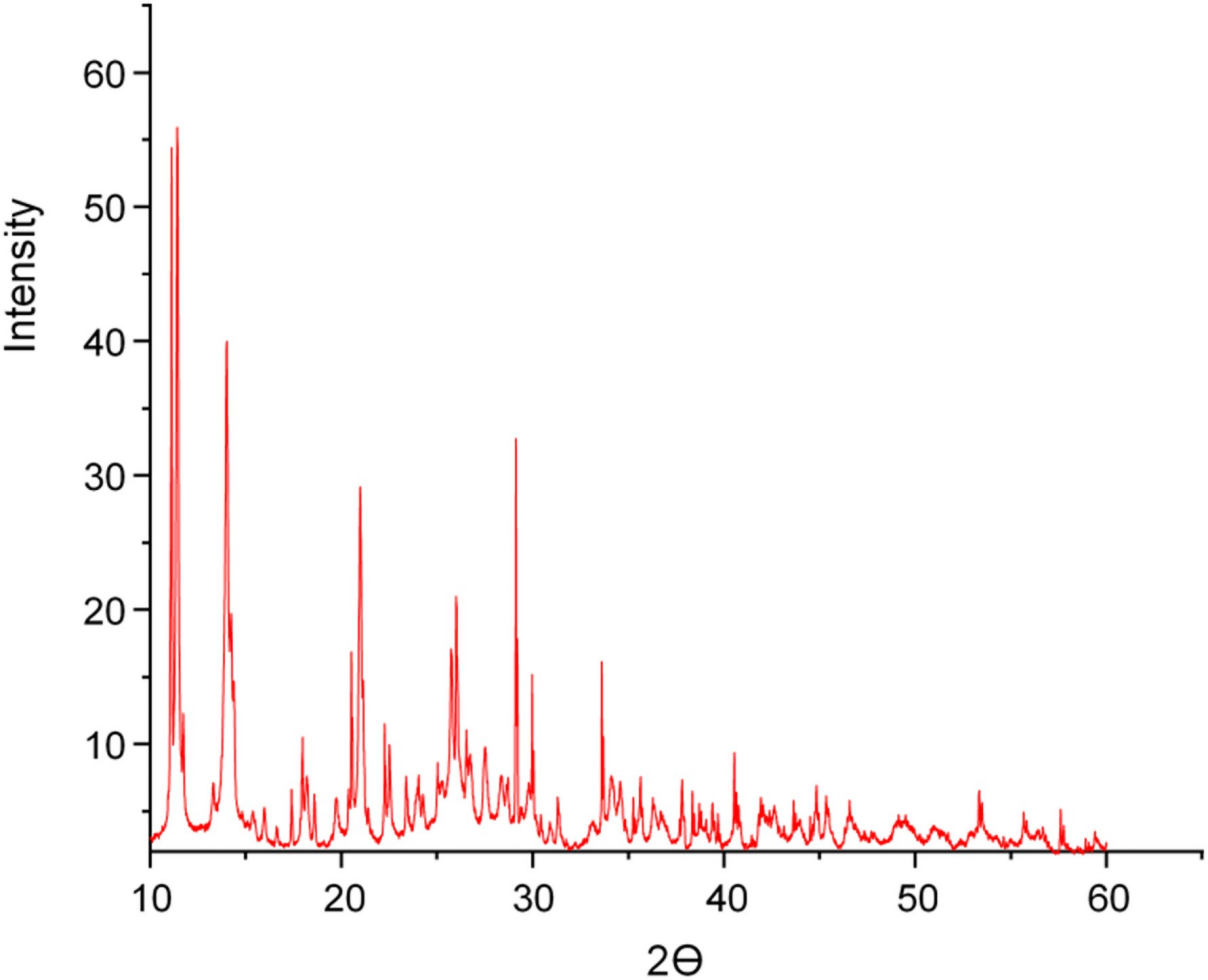
X-ray powder diffraction patterns of the [(UO_2_)(OAc)_2_(MP)] complex.

To obtain evidence of the structure of the uranyl complexes, an X-ray powder diffraction study was performed. The complex [(UO_2_)(OAc)_2_(SCZ)] and [(UO_2_)(OAc)_2_(MP)] showed a crystalline nature with very thin diffraction peaks, which imply a long-range ordering of the two materials [34]. The X-ray diffractogram of the (UO_2_^2+^) complexes, Fig 5 and S1 Fig, show reflecting peaks in the range of 2θ from 10° to 60°. The estimated crystallite sizes were calculated according to Debye-Scherrer formula; $Dhkl=\frac{k\lambda }{\beta cos\theta }$ where k is a constant value 0.94, the wavelength of X-ray used (λ = 0.154 nm) and β is the full width at half maxima of all peaks of the XRD patterns FWHM and θ is Bragg angle [35]. The crystalline size was found for [(UO_2_)(OAc)_2_(SCZ)] and [(UO_2_)(OAc)_2_(MP)] complex 62.91 nm and 121.2 nm, respectively.

### 3.7. Thermal analysis (TGA)

**Fig 6:**
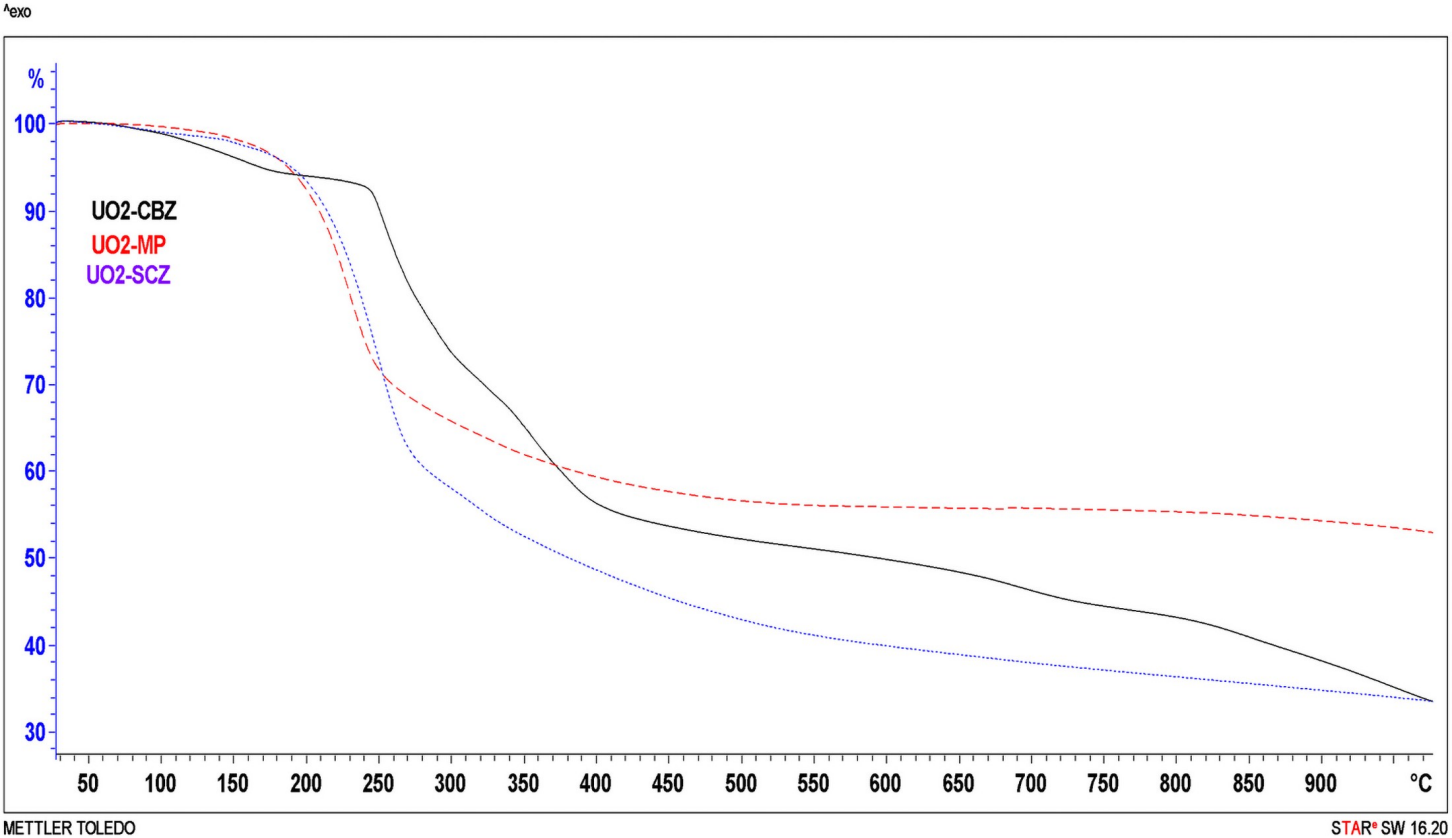
TGA curves for the uranyl complexes in the range 25–1000°C.

The new complexes were subjected to TGA to study their coordination structure [36, 37]. The TGA curves for the synthesized complexes are presented in Fig 6.

[(UO_2_)(OAc)_2_(CMZ)] showed two decomposition steps, starting at the dehydration range (150–220°C) and losing coupled water molecules. The final stage ended by organic molecule elimination with 39.6% (cal. = 40.4%) of the sample weight. The final solid-state is (UO_3_) and calculated carbon atoms.

The [(UO_2_)(OAc)_2_(SCZ)] and [(UO_2_)(OAc)_2_(MP)] curves exhibited a one-step pyrolysis process that started at 176 and ended at 245°C with a mass loss of 55.5% (cal.55.2%) and 40.9% (cal.41%), respectively. The remaining product is UO_3_ formed as a metal residue. S2 Table shows the percentage assignments for each step, and the calculated values.

These curves were applied to compute kinetic parameters using two methods, the Coast-Redfern integral [22] and the approximation of Horowitz-Metzger [23] method. Moreover, thermodynamic parameters were determined by applying the following equations; [37, 38] the activation enthalpy ΔH = E-RT, activation entropies ΔS = R[In(Ah/kT)], and Gibbs free energy ΔG = ΔH-TΔS, where k is the Boltzmann’s constant and h is the Planck’s constant. Their values for each step are summarized in S3 Table, and the drawn relation of both methods is presented for the individual complexes in S4 Table. The negative values of the activation entropies (ΔS) indicating the degradation operations are slow due to the activated complex being more ordered than the reactants [37, 39]. The positive values of ΔH and ΔG illustrated that the reactions were endothermic and endergonic.

### 3.8. Optical properties

UV–vis spectroscopy was performed to study the effect of the metal ions on the optical properties after coordination to the ligand. The UV-Vis absorption spectrum in 10^−3^ M DMSO solution is presented in S2 Fig, which shows the main absorption peak for [(UO_2_)(OAc)_2_(CMZ)], [(UO_2_)(OAc)_2_(MP)], and [(UO_2_)(OAc)_2_(SCZ)]. The peak that appeared in the CMZ spectrum at 256 nm is assigned to π→π* transitions [40] and red shifted to 266 nm for the formed [(UO_2_)(OAc)_2_(CMZ)] complex. Moreover, a new peak between 400–500 nm in the metal complexes spectra could be assigned to LMCT phenomena [41]. The 6-MP spectrum exhibited a band at 270 cm^-1^ assigned to the π-π* transition originating from the aromatic ring. This band shifted to a lower frequency (268 nm) after coordination to the metal ion. Similarly, the free SCZ ligand has a band in 276 nm assigned to the n→π transition and maintained in the metal spectrum. Several uranyl complexes showed weaker bands that can be not distinguished easily at low concentrations, although a band at 447 nm in the [(UO_2_)(OAc)_2_(SCZ)] was observed and could arise from partially forbidden charge transfer transitions to non-bonding, unoccupied f-orbitals [42].

### 3.9. Biological studies

#### 3.9.1 In vitro DNA binding

**Fig 7:**
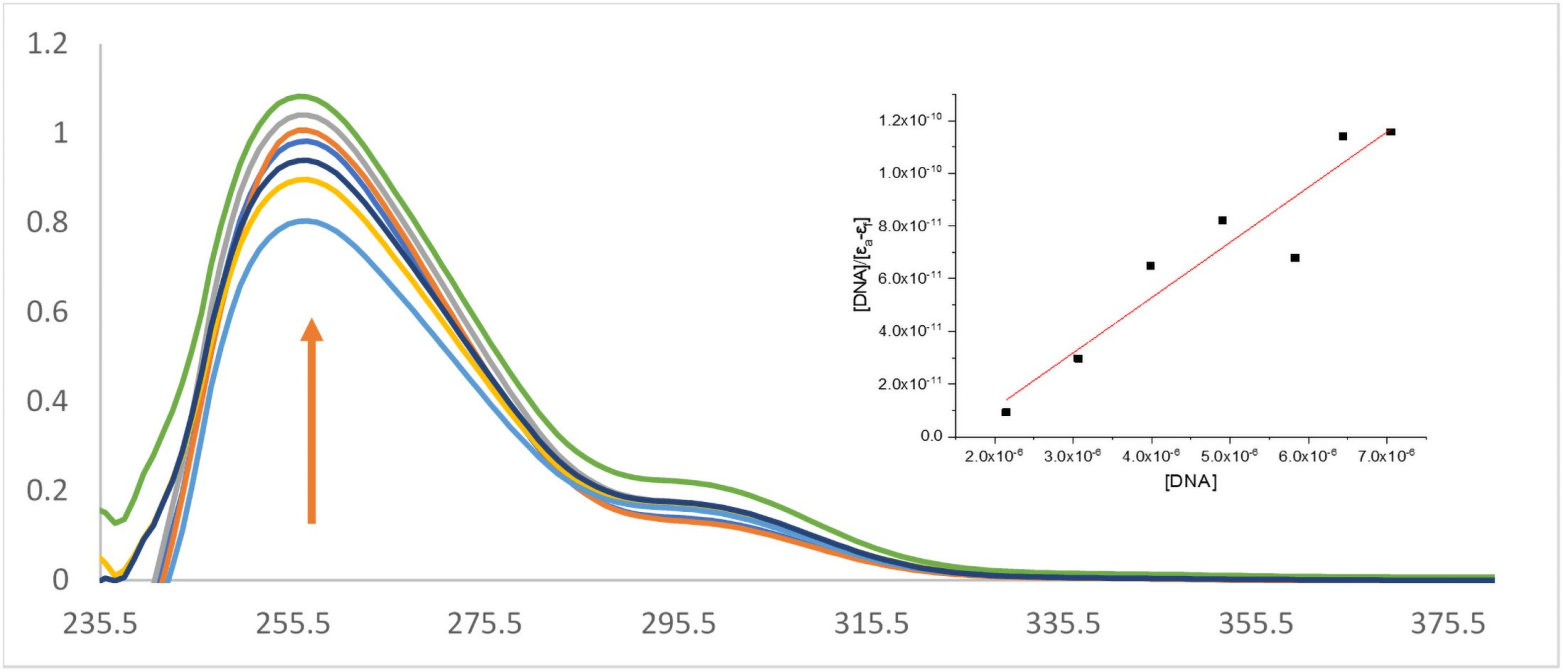
The hyperchromic reactions of [(UO_2_)(OAc)_2_(SCZ)] (arrow indicates changes with increasing DNA concentration).

*3*.*9*.*1*.*1 Absorption titrations*. The binding ability of the prepared samples was evaluated by UV absorption by measuring the effects of adding various concentrations of CT-DNA to the metal complexes. The maximum absorption band for the free ligands was in the range 263–265 nm that had a blue shift after binding to DNA. The metal complexes also showed this blue shift. The metal complexes have a hyperchromic effect on the molar absorptivity, Fig 7 and S3 Fig, confirming that binding to the DNA double-helix results in changes in its structure [43, 44]. The hyperchromic effect revealed denaturation of the DNA helix during the binding process [18, 45]. The calculated binding constants were estimated to be K_b_ = 4.3 ×10^5^ M^-1^, 5×10^5^ M^-1^ and 6.9 ×10^5^ M^-1^ for uranyl with CMZ, MP, and SCZ metal complexes, respectively.

**Fig 8:**
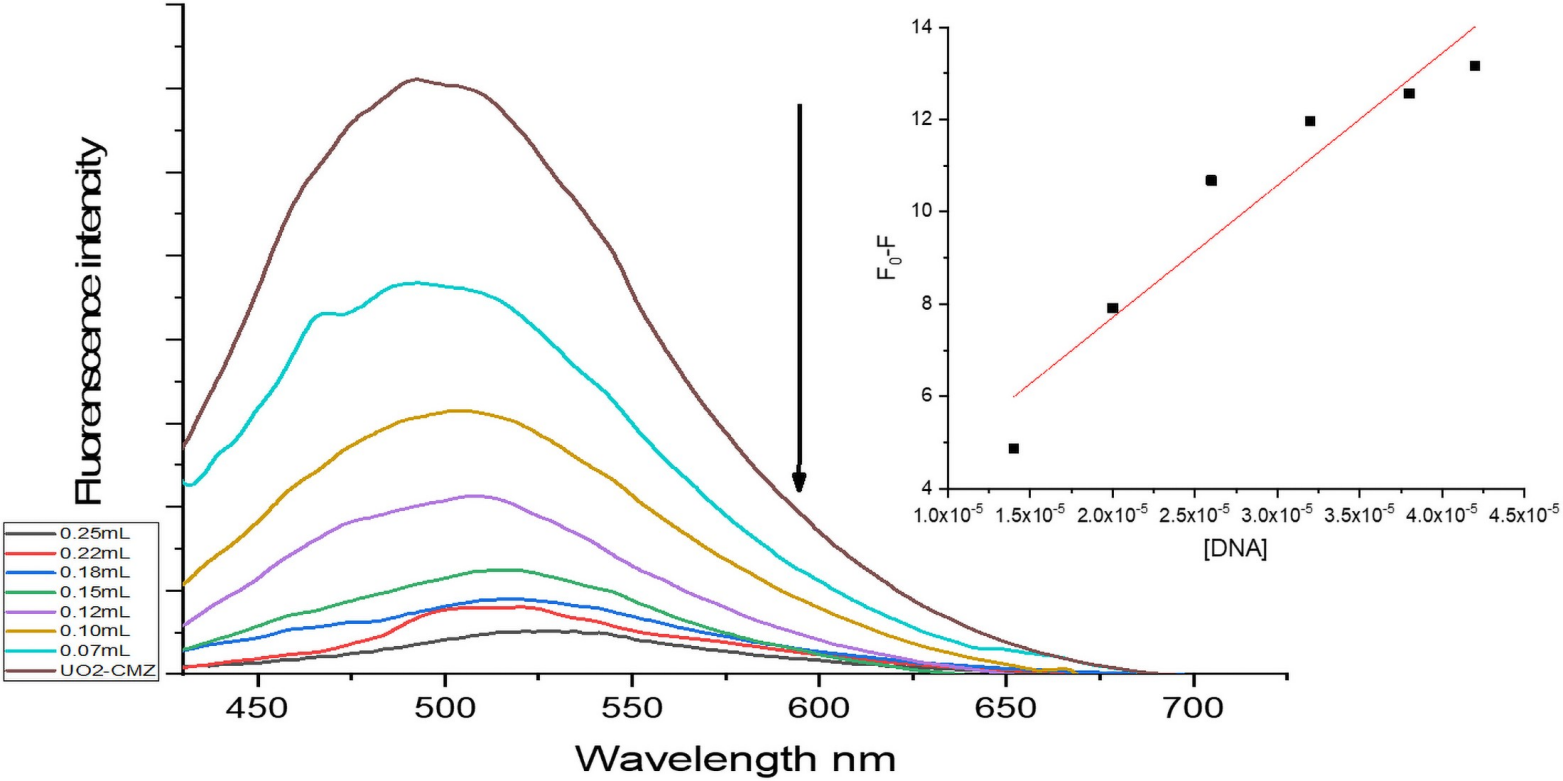
Emission spectra of [(UO_2_)(OAc)_2_(CMZ)] complex in Tris–HCl buffer in the absence and presence of CT–DNA. The arrow shows the intensity change upon increasing CT–DNA concentrations.

*3*.*9*.*1*.*2 Fluorescence spectroscopic studies*. The uranyl complexes exhibited luminescence either in DMSO or in the presence of CT–DNA. Therefore, we tested the binding of the complexes to DNA by fluorescence spectral titration in the absence of any probes. A constant concentration of the metal complexes (1×10^5^M) was titrated with increasing concentrations of DNA from 1.4 ×10^5^ to 4.6×10^5^M. When the complex of [(UO_2_)(OAc)_2_(CMZ)] exited at 340, Fig 8, the intensity of emission appreciably decreased with increasing DNA concentrations and with a red shift in the position of the emission bands from 500 nm to 515nm. This quenching could be attributed to charge transfer from the guanine base of DNA to the orbitals of the complex due to the partial or complete interaction with double-stranded DNA. Moreover, the binding results in shielding of the complex by the DNA helix from solvent molecules, leading to a decrease in the vibrational mode of relaxation [46]. Cation metal ions exert a strong electrostatic attraction to the anionic phosphate backbone of DNA, and form non-covalent bonds that results in a so-called groove binding mode [18].

Both [(UO_2_)(OAc)_2_(MP)] and [(UO_2_)(OAc)_2_(SCZ)], S4 Fig, showed enhanced emission intensity when excited at 390 and 450 nm, respectively. Additionally, an observed shift from 430 to 438nm for [(UO_2_)(OAc)_2_(MP)] and 430 to 433nm for [(UO_2_)(OAc)_2_(SCZ)] could be caused by the environment change to non-polar due to the bonding of the metal complex to DNA [47]. The rotation of the free uranyl molecules is always preferred to radiationless decay of the excited states in the case that they bind to DNA then the deactivation will be through fluorescence emission, resulting in a significant increase in the fluorescence intensity [18].

The Scatchard equation [14] was used to determine the binding constant K_b_ for the tested complexes, which were 2.9×10^5^, 1.6×10^5^ and 2.4×10^4^ M^-1^ for [(UO_2_)(OAc)_2_(CMZ)], [(UO_2_)(OAc)_2_(MP)], and [(UO_2_)(OAc)_2_(SCZ)], respectively.

#### 3.9.2 In vitro binding studies with HSA

**Fig 9:**
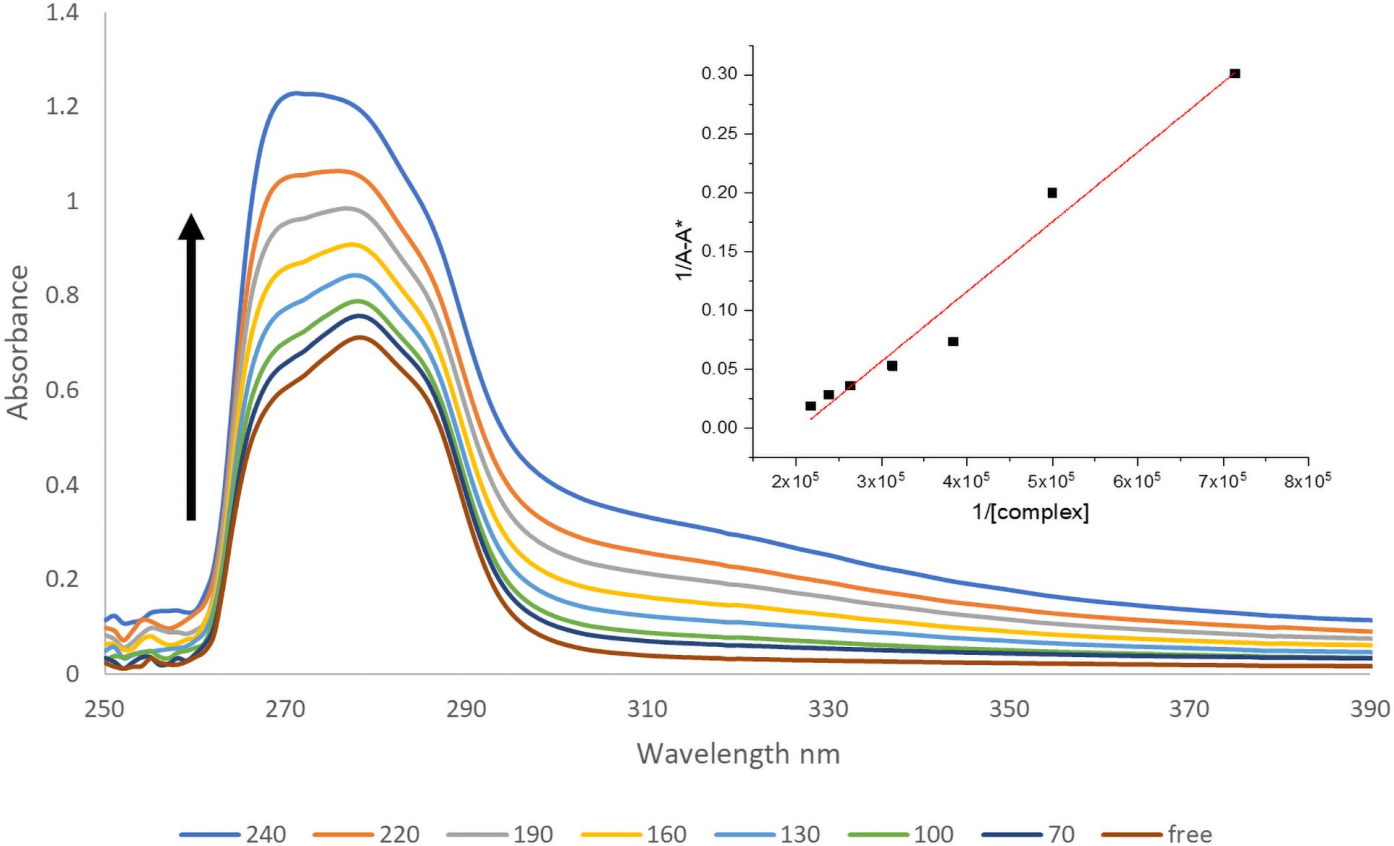
Absorption spectra of HSA in the absence and presence of increasing amounts of [(UO_2_)(OAc)_2_(MP)] complex, indicated by the arrow.

*3*.*9*.*2*.*1 Electronic absorption studies*. The interaction between HSA and the prepared complexes was evaluated using electronic absorption spectroscopy. The free HSA λ_max_ absorption at 279 nm is attributed to the protein chromophores, phenylalanine, tyrosine, and tryptophan. The gradual addition of the uranyl complexes, 1.4×10^−5^ to 4.6×10^−5^ M, to the constant concentration of HSA (2.4×10^−5^ M), presented a sharp increase in the absorption intensity. This hyperchromic mode was associated with the varied shift of the λ_max_, [(UO_2_)(OAc)_2_(CMZ)], S5 Fig, and [(UO_2_)(OAc)_2_(SCZ)] complexes showed a red shift to long wavelengths, 287 nm and 282nm, respectively, which indicates strong binding of the metal complexes due to the morphological changes in the secondary structure of HSA. The binding was predominantly electrostatic interactions and could be facilitated by hydrogen bond formation [46]. In contrast, [(UO_2_)(OAc)_2_(MP)] showed a blue shift to 272 nm, Fig 9, revealing that the non-covalent interaction leads to a change of the absorption profile initiating from the effect of the water solvent, and tends to disturbance in the micro-environment of the polypeptide backbone of the HSA.

The calculated intrinsic binding constant (K_b_) of the investigated complexes can be arranged in the order, 2.26×10^5^, 2.10×10^5^, and 7×10^4^ for MP, CMZ, and SCZ uranyl complexes, respectively. The obtained values suggested strong binding of MP and CMZ metal complexes to HSA, while SCZ has only moderate binding.

**Fig 10:**
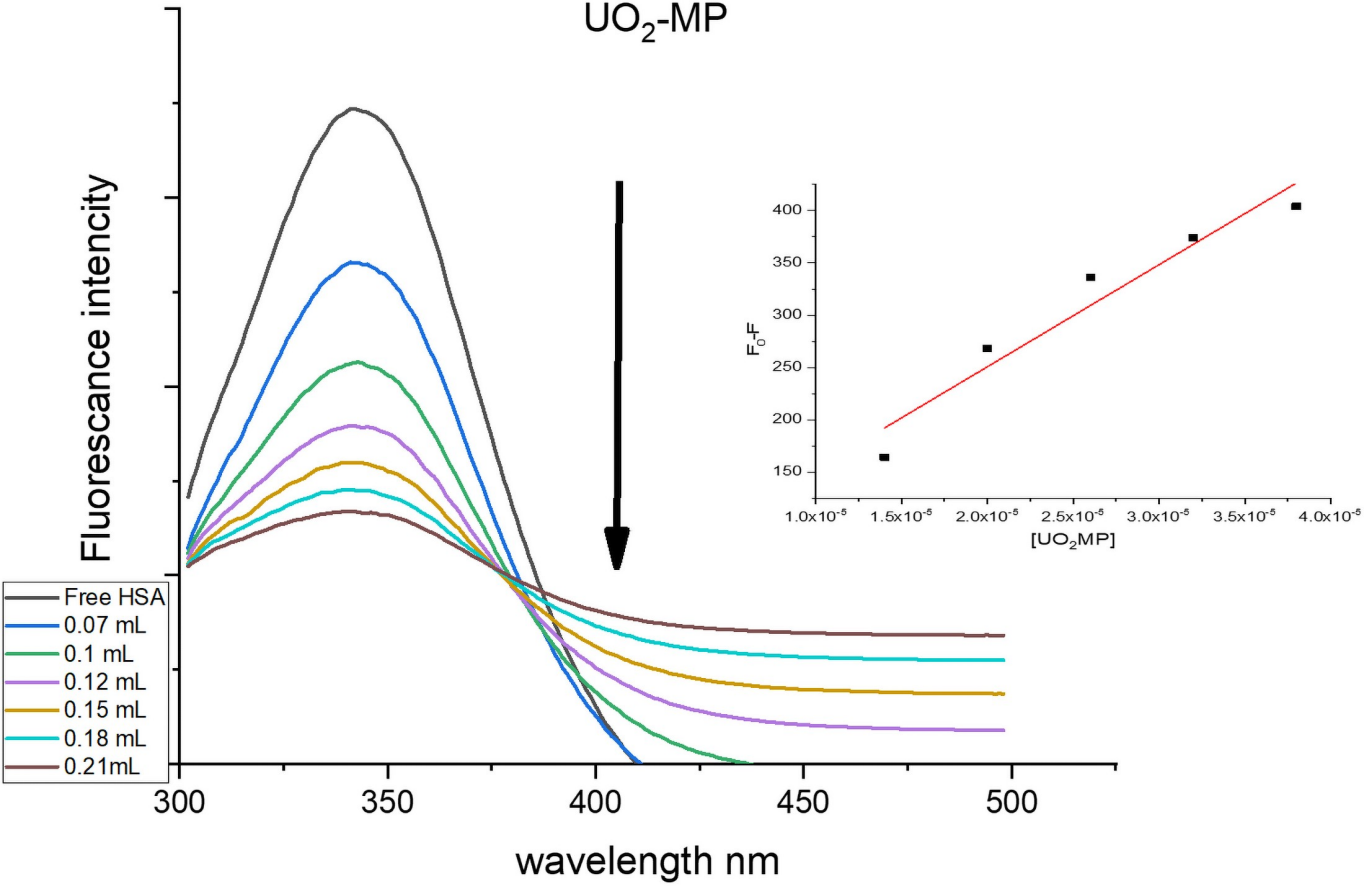
Emission spectrum of [(UO_2_)(OAc)2(MP)] complexes bound to HSA in the presence and absence of the complexes. Inset shows the plots of emission intensity I_0_/I vs. [Complex] for determining K_SV_.

*3*.*9*.*2*.*2 Fluorescence interaction studies*. Fluorescence spectroscopy was used to gain insight into the quenching mechanism of the HSA and the uranyl complexes. The fluorescence of HSA is attributed to the amino acid residue tryptophan (Trp), which is located in subdomain IIA of the HSA structure [40]. The interaction between the indole moiety of the Trp chromophore can change the fluorescence intensity of HSA. The effect of a gradual increase of the uranyl complexes showed a quenching of the HSA fluorescence emission at 344 nm. The [(UO_2_)(OAc)_2_(CMZ)] complex showed a blue shift to 334 nm, which indicates an interaction with HSA, S6 Fig. Similarly, the SCZ complex was shifted to low frequency, 339 nm (S6 Fig), while no shift was observed in the emission wavelength for the [(UO_2_)(OAc)_2_(MP)] complex. The K_SV_ values could be ordered incrementally, 9.75×10^6^, 9.46×10^6^, 7.2×10^6^ for [(UO_2_)(OAc)_2_(MP)], [(UO_2_)(OAc)_2_(SCZ)], and [(UO_2_)(OAc)_2_(CMZ)], respectively. The data correlated with the absorbance results, i.e., that [(UO_2_)(OAc)_2_(MP)] binds strongly to HSA. The fluorescence spectra of HSA in the absence and presence of the [(UO_2_)(OAc)_2_(MP)] complex as a quencher in Tris–HC1 buffer (pH 7.4) is shown in Fig 10.

#### 3.9.3 Antibacterial activity

**Fig 11:**
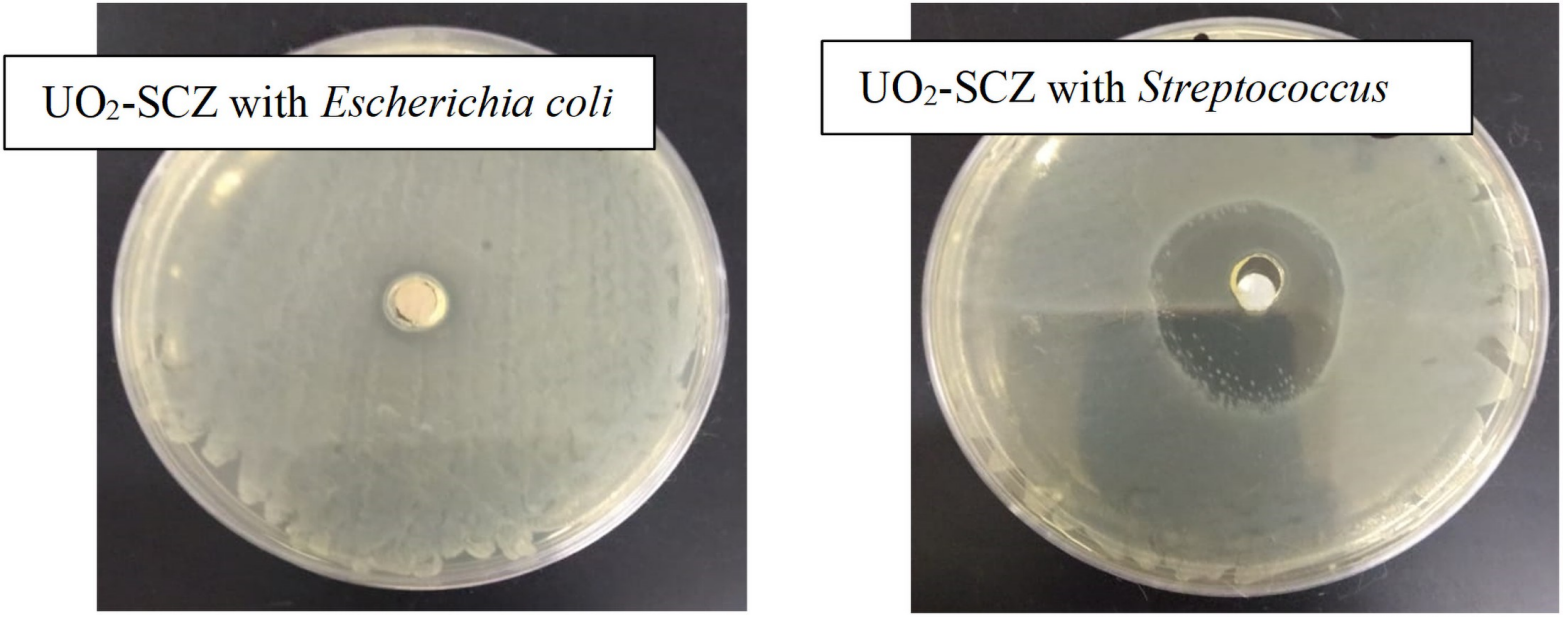
Antibacterial activity of the synthesized (UO2)(OAc)2(SCZ)] with gram-negative bacteria (*Escherichia coli*) and gram-positive bacteria (*Staphylococcus aureus*).

In vitro screens of the synthesized uranyl complexes were carried out to evaluate their bioefficacy against the growth of various bacteria. Two gram-negative bacterial strains; *Escherichia coli* (*E*.*coli*) and *Klebsiella pneumonia*, and two gram-positive bacterial strains; *Staphylococcus aureus* (*S*. *aureus*) and *Streptococcus mutans* were chosen for this investigation. Two standard antibiotics were employed to evaluate the results: Gentamicin, which is used against Gram negative bacteria, and Ampicillin, which is used against Gram positive bacteria. The results are expressed as Mean ± Standard deviation (mm), and are illustrated in Table 6. The agar disk diffusion test graphics are shown in Fig 11. The synthesized complexes showed inhibition against *Klebsiella pneumonia*, and [(UO_2_)(OAc)_2_(MP)] showed the highest effective inhibition (28.3 mm), even more than Gentamicin (25mm). Additionally, a similar degree of inhibition, ranging between 10–16 mm for the metal complex, was shown against *E*.*coli*. On the other hand, the [(UO_2_)(OAc)_2_(CMZ)] complex showed substantial antibacterial activity against *Streptococcus mutans* and *S*. *aureus* with 28.6 mm and 18.6 mm, which are close to the values of Ampicillin, i.e., 30 mm and 22mm, respectively. The uranyl complexes were found to be more active against *Klebsiella pneumonia* and *Streptococcus mutans*. This could be due to the chelation to the metal ion, which improves the free drug’s ability to permeate the lipid layer of the microorganisms [9].

**Table 6:** Antibacterial activity of the synthesized uranyl complexes by agar cup method.

Sample Microorganism	[(UO_2_)(OAc)_2_(CMZ)]	[(UO_2_)(OAc)_2_ (MP)]	[(UO_2_)(OAc)_2_(SCZ)]	Standard antibiotic
Gram-negative bacteria				**Gentamicin**
***Escherichia coli (ATCC*:*10536)***	10.3±0.5	15.6±0.5	11.3±0.5	27±0.5
***Klebsiella pneumonia (ATCC*:*10031)***	20.3±0.6	28.3±0.6	24.6±0.6	25±0.5
Gram-positive bacteria				**Ampicillin**
***Staphylococcus aureus (ATCC*:*13565)***	18.6±0.6	15.6±0.5	16.6±0.5	22±0.1
***Streptococcus mutans (ATCC*:*25175)***	28.6±0.6	17.3±0.5	26.6±0.6	30±0.5

#### 3.9.4 In vitro cytotoxicity assay

The cytotoxic effects of the metal complexes were examined against three different cancer cell lines, MCF7 (breast cancer cell line), SK-MM-1 (myeloma cell line) and Caco-2 (Colon cell line), over 24h by adding medium containing the metal complexes at varying concentrations (10–500 μg/ml), Table 7. Generally, the uranyl complexes were more cytotoxic than the free organic drugs. The obtained IC_50_ values for the metal complexes exhibited moderate to weak inhibition toward the cancer cell lines. Notable cytotoxicity was observed with the [(UO_2_)(OAc)_2_(SCZ)] complex on the colon cancer cells while MP- UO_2_ complex showed sufficient efficiency on the myeloma cells with IC_50_ = 55.6 μM. Comparing the molecular structure of the investigated complexes, we can obseve that MP- UO_2_ and SCZ- UO_2_ have a pyrimidine ring which could rise the potency of the complexes. The attributes of pyrimidines as anticancer agent are the most extensively reported in literature [41]. In contrast, the [(UO_2_)(OAc)_2_(CMZ)] complex had very limited cytotoxic effects on the cell lines.

**Table 7:** Cytotoxic activity of the three free drugs and their metal complexes against three human tumor cell lines.

Compound	*In vitro* Cytotoxicity IC_50_ (μg/ml)^a^		
	Breast cell line (Mcf7)	myeloma cell line (SK-MM-1)	Colon cell line (Caco-2)
CMZ	313.767	-	103.477
[(UO_2_)(OAc)_2_(CMZ)]	88.6	-	177.27
MP	-	204.911	98.79
[(UO_2_)(OAc)_2_(MP)]	-	55.6	103.3
SCZ	215.24	-	97.6
[(UO_2_)(OAc)_2_(SCZ)]	106.87	-	42.15

^a^- IC_50_ (mg/ml): 1–10 (very strong), 11–20 (strong), 21–50 (moderate), 51–100 (weak) and above 100 (non-cytotoxic).

## 4. Conclusion

Three new bidentate metal complexes of uranyl were synthesized and fully characterized by spectroscopies, and thermogravimetric methods. The IR analysis indicated that the linearity of the U = O and the bond lengths were in the range 1.734–1.779 A°. The difference between the frequency of υ_asy_(OCO) and υ_s_(OCO) of the acetate group revealed that acetate has monodentate binding to the metal ion. The optimized geometries revealed that the complexes have distorted octahedral geometry around the uranium central atom where the metal ion binds to acetate by monodentate binding. Molecular electrostatic potential maps showed the susceptibility of the acetate group to nucleophilic attack, while the ligand tended to be electrophilic sites. The electrophilicity values assumed that [(UO_2_)(OAc)_2_(SCZ)] and [(UO_2_)(OAc)_2_(MP)] have practical biological activity. The DNA binding ability of the UO_2_^2+^ complexes was tested by two techniques, and showed that [(UO_2_)(OAc)_2_(SCZ)] and [(UO_2_)(OAc)_2_(CMZ)] have the highest binding constant (K_b_). Strong binding for MP and CMZ metal complexes with HSA were observed by both absorbance and fluorescence methods, while [(UO_2_)(OAc)_2_(SCZ)] showed only moderate binding. The anti-microbial activity of the synthesized complexes were also investigated and showed that [(UO_2_)(OAc)_2_(MP)] has a higher inhibition effect than the standard antibiotic of gram-negative bacteria, Gentamicin, against *Klebsiella pneumonia*. [(UO_2_)(OAc)_2_(CMZ)] worked more effectively against *Streptococcus mutans*, a gram-positive bacteria, than the other tested complexes. In vitro cytotoxicity towards three human cancer lines showed a moderate inhibition for [(UO_2_)(OAc)_2_(SCZ)] and [(UO_2_)(OAc)_2_(MP)]. We conclude that uranyl ions can improve the antibacterial and anticancer properties of the free ligand.

## Supporting information

S1 Fig X-ray powder diffraction patterns of UO_2_-SCZcomplex. (TIF)

S2 Fig UV spectra for CMZ, MP and SCZ UO_2_(II) complexes. (TIF)

S3 Fig The hyperchromic reactions of (a) [UO_2_(CMZ)(ACO)_2_], (b) [UO_2_(MP)(ACO)_2_] (arrows indicate changes with increasing DNA concentration). (TIF)

S4 Fig Emission spectra of UO_2_(II) complex in Tris–HCl buffer the absence and presence of CT–DNA. Arrow shows the intensity change upon increasing CT–DNA concentration.
(TIF)

S5 Fig Absorption spectra of HAS in the absence and presence of increasing amount of UO_2_ complex indicates by an arrow. (TIF)

S6 Fig Emission spectrum of UO2(II) complexes bound to HAS in the presence of the complexes and in absence of the complexes. Inset shows the plots of emission intensity I0/I vs. [complex] for determining KSV.
(TIF)

S1 Table Gaussian parameters using B3LYP/6–11 G and SDD method. (DOCX)

S2 Table Thermal data for UO_2_(II) complexes. (DOCX)

S3 Table Kinetic data of metal complexes using Coats-Redfern and Horowitz and Metzger equation. (DOCX)

S4 Table Horowitz–Metzger (HM) and Coats–Redfern (CR) of metal complexes. (DOCX)
