# Supplementary figures and images for: Complexation of uranyl (UO2)2+ with bidentate ligands: XRD, spectroscopic, computational, and biological studies
Source: PLoS One. 2021 Aug 19;16(8):e0256186. doi: 10.1371/journal.pone.0256186 (PMC8376047; doi:10.1371/journal.pone.0256186)

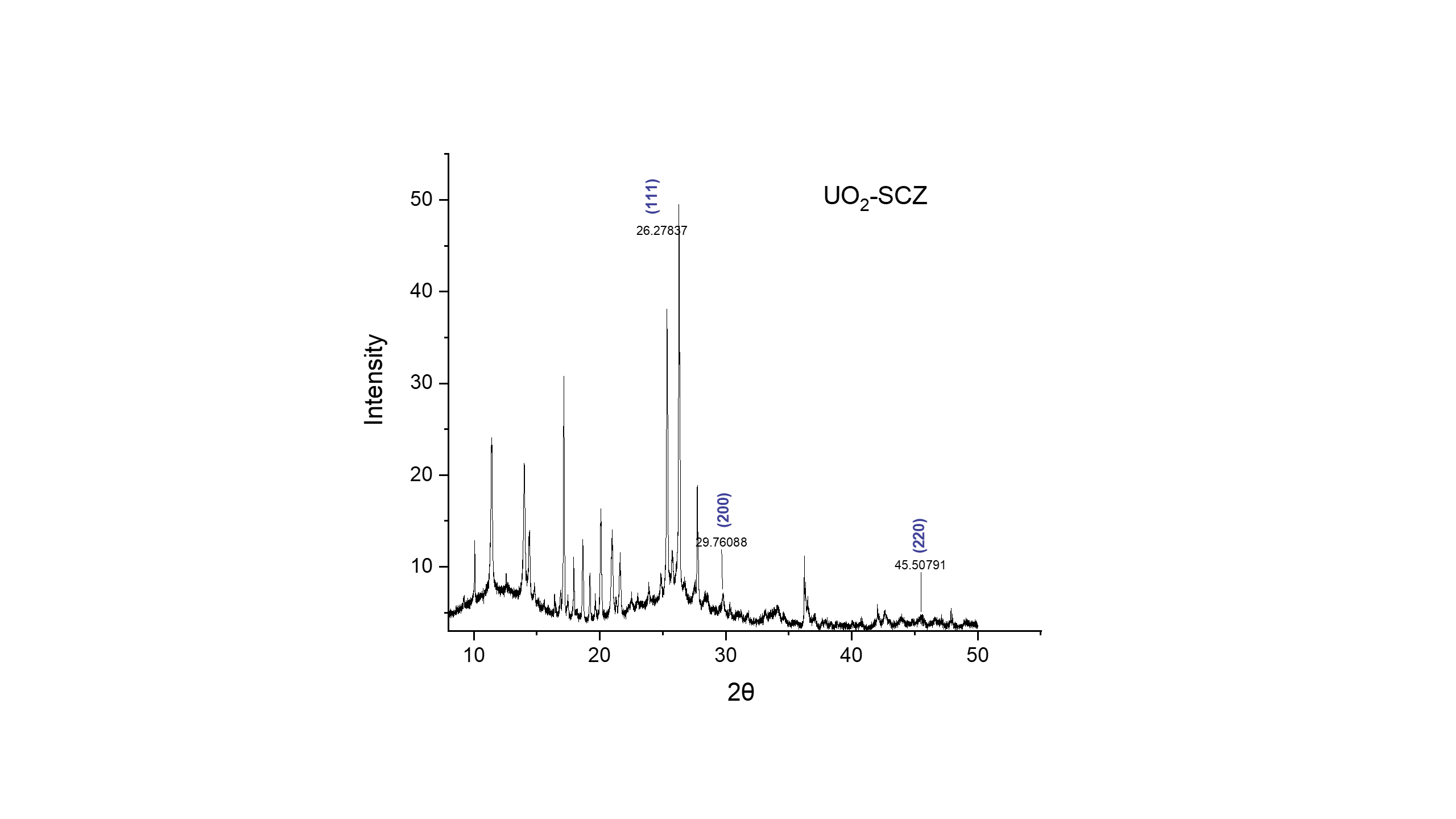

Supplement: S1 Fig — (TIF) [file pone.0256186.s001.tif]

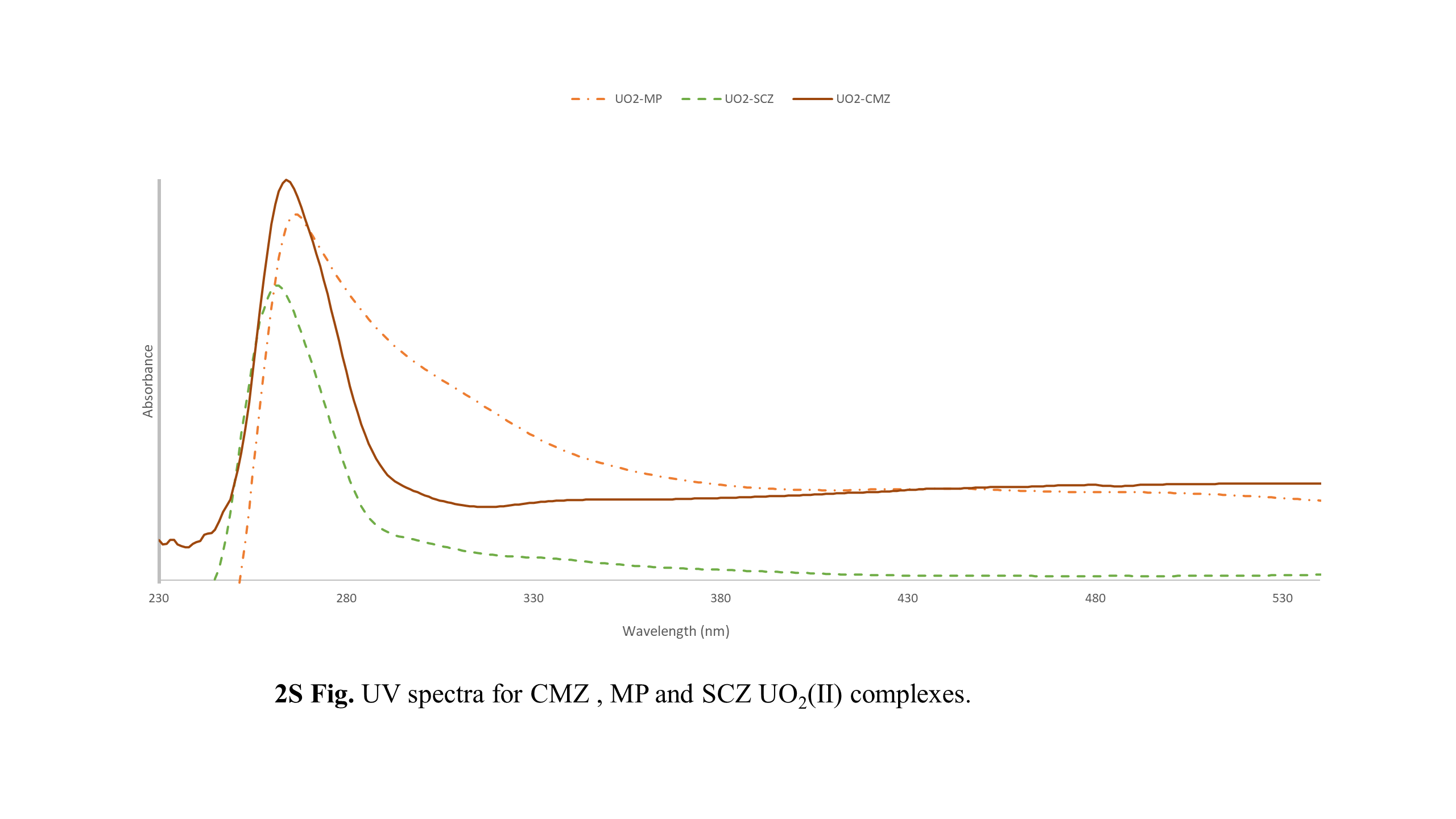

Supplement: S2 Fig — (TIF) [file pone.0256186.s002.tif]

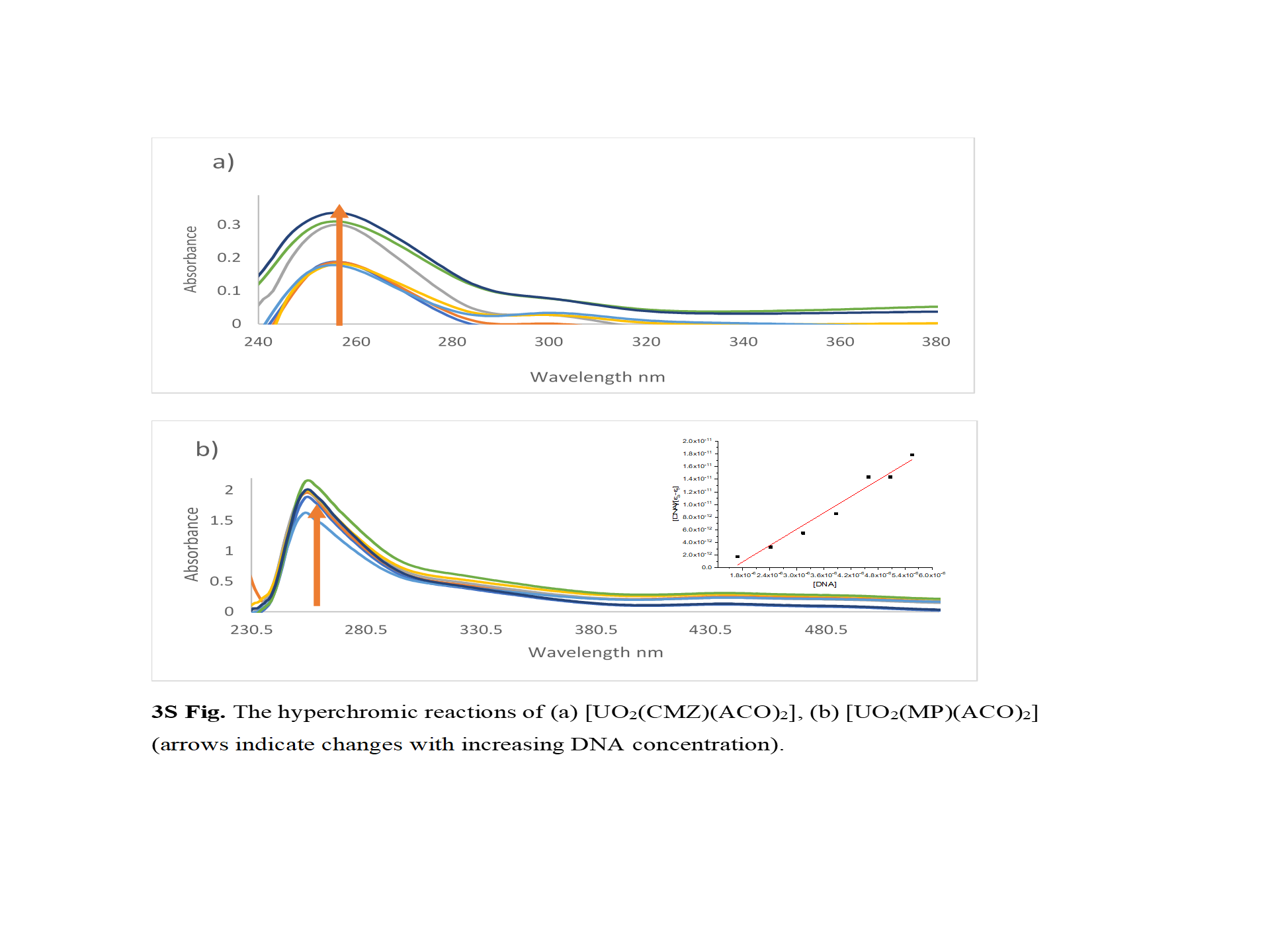

Supplement: S3 Fig — (TIF) [file pone.0256186.s003.tif]

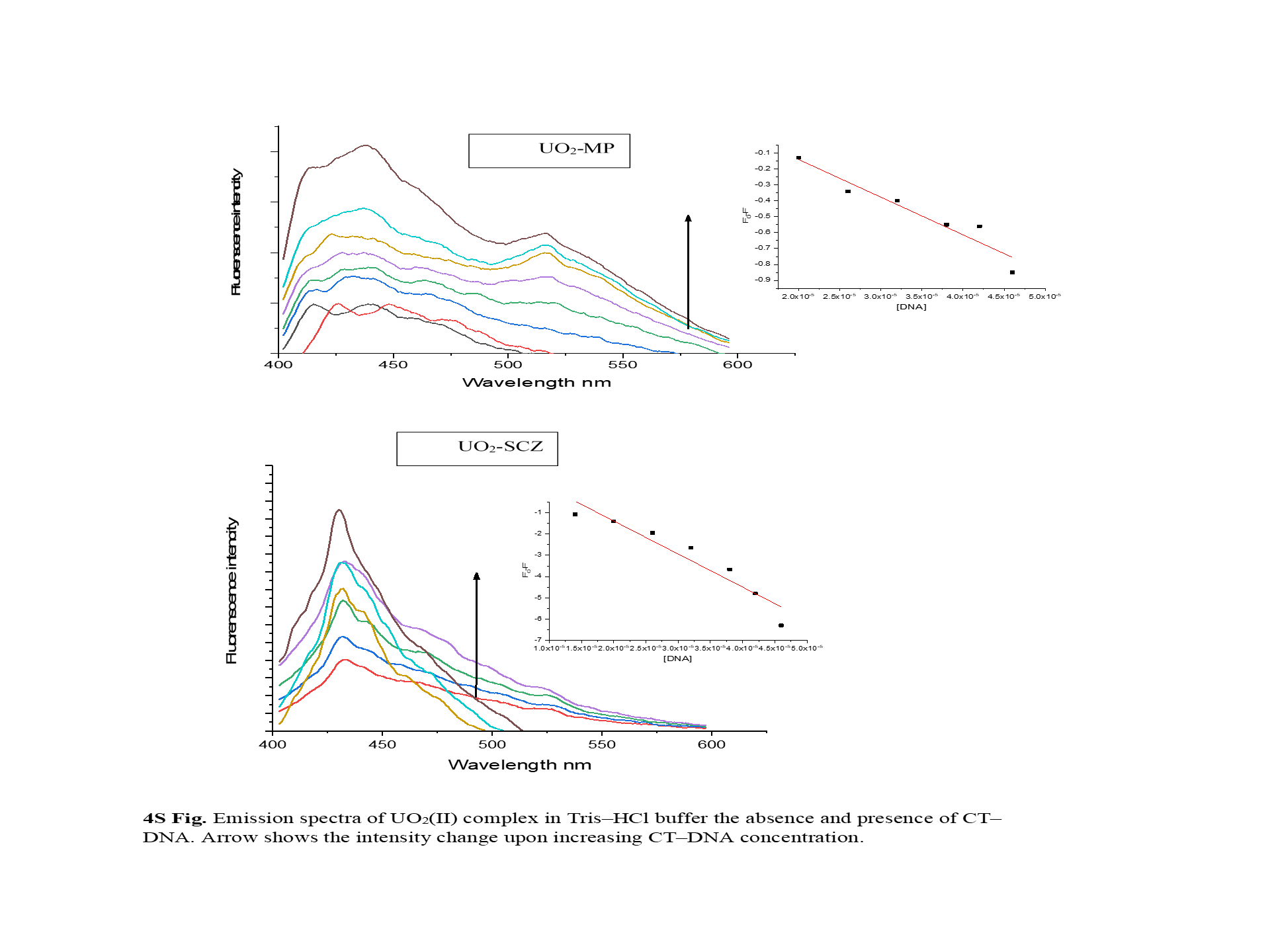

Supplement: S4 Fig — Arrow shows the intensity change upon increasing CT–DNA concentration. (TIF) [file pone.0256186.s004.tif]

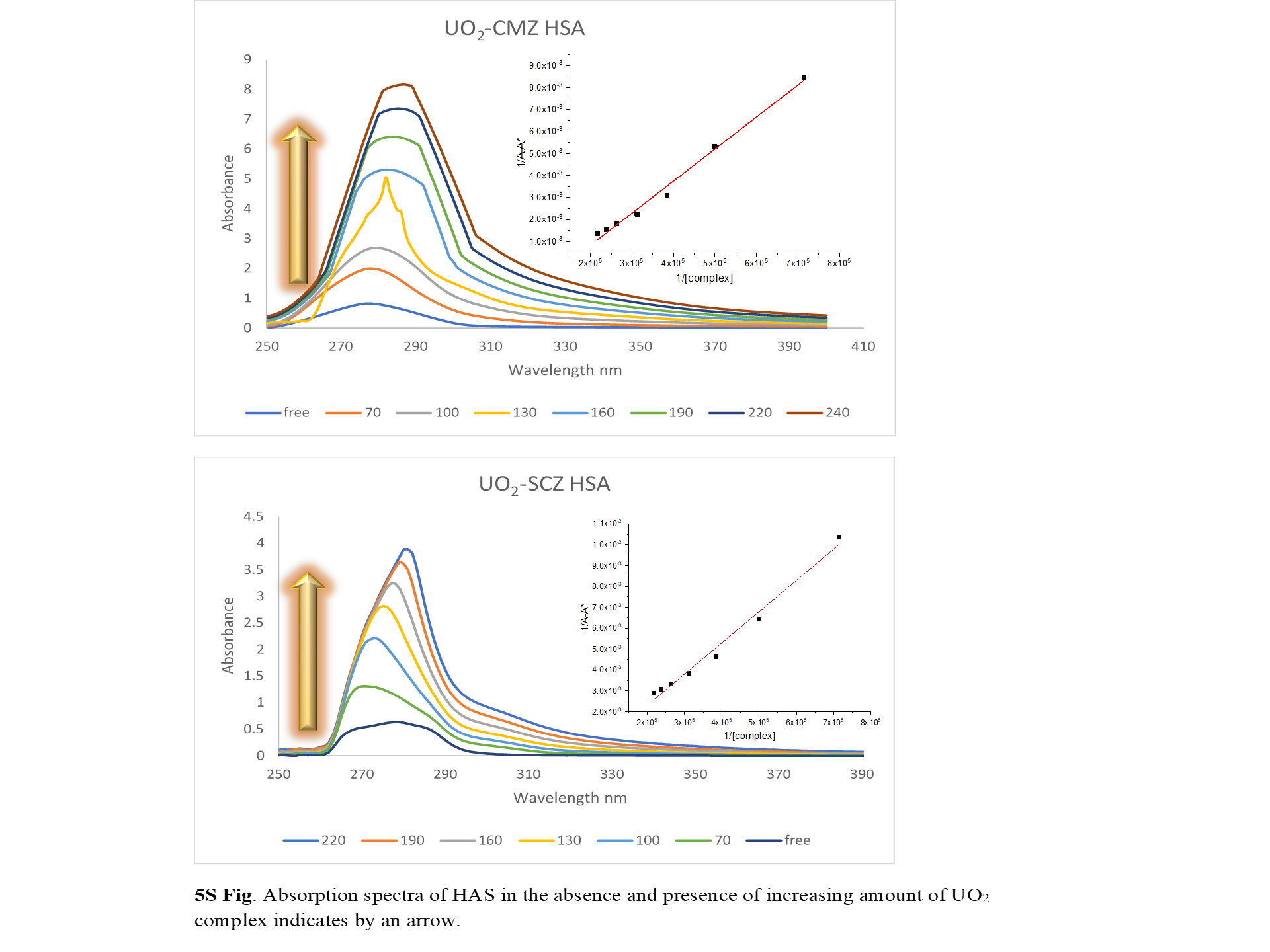

Supplement: S5 Fig — (TIF) [file pone.0256186.s005.tif]

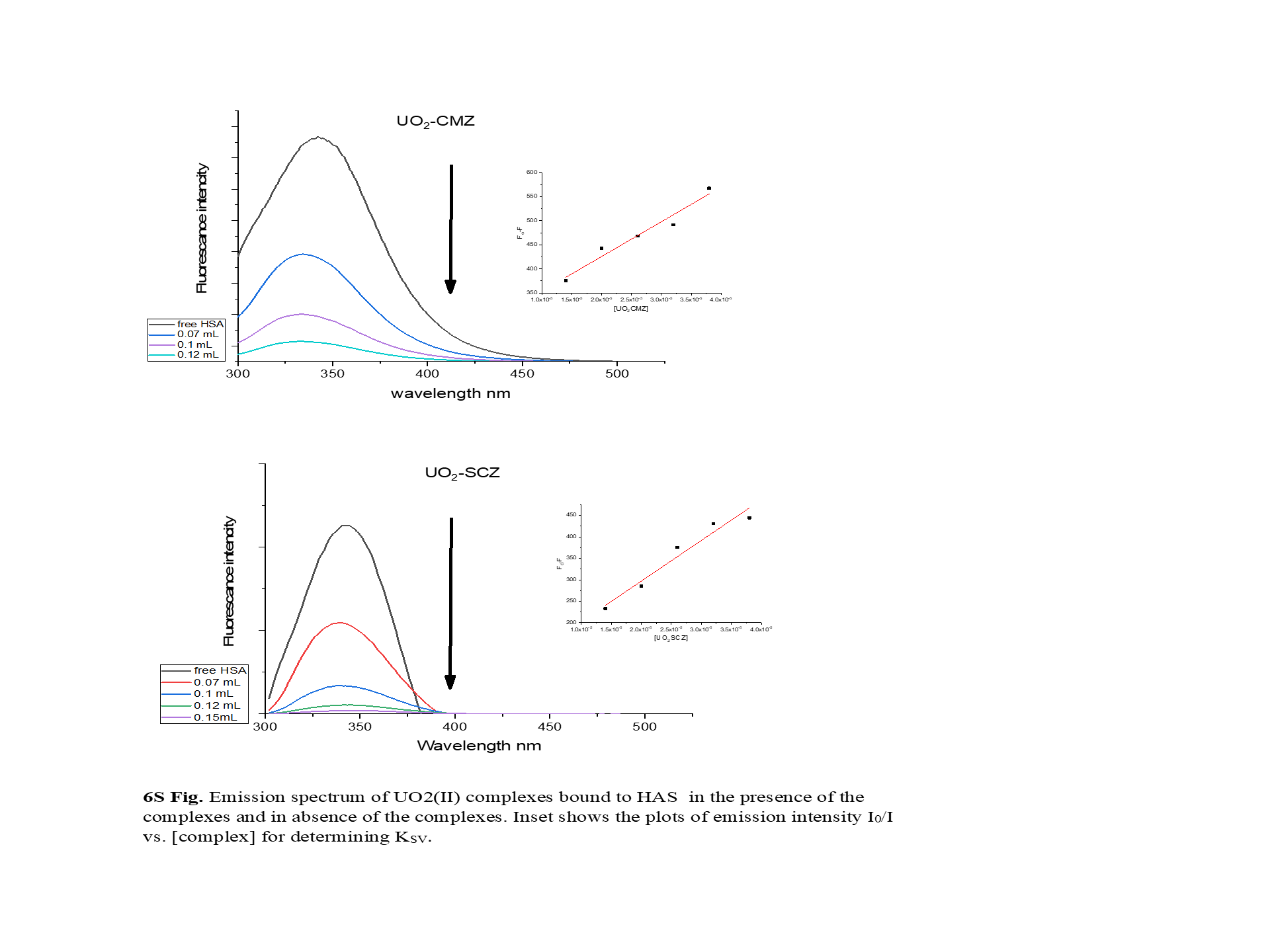

Supplement: S6 Fig — Inset shows the plots of emission intensity I0/I vs. [complex] for determining KSV. (TIF) [file pone.0256186.s006.tif]
