# Supplementary material for: Complexation of uranyl (UO2)2+ with bidentate ligands: XRD, spectroscopic, computational, and biological studies
Source: PLoS One. 2021 Aug 19;16(8):e0256186. doi: 10.1371/journal.pone.0256186 (PMC8376047; doi:10.1371/journal.pone.0256186)
Supplement: S1 Table — (DOCX) [file pone.0256186.s007.docx]

**S1 Table .** Gaussian parameters using B3LYP/6–11 G and SDD method.

| **compound** | **HUMO** | **LUMO** | **∆ E** | **x** | **ɳ** | **σ** | **Pi** | **σ** | **S** | **ω** | **ΔN max** |
| --- | --- | --- | --- | --- | --- | --- | --- | --- | --- | --- | --- |
| CMZ | -1.00 | -5.55 | -4.55 | 3.28 | -2.28 | -0.44 | -3.28 | -0.44 | -1.14 | 2.36 | -1.44 |
| UO_2_-CMZ | -6.85 | -3.03 | 3.82 | 4.94 | 1.91 | 0.52 | -4.94 | 0.95 | 0.95 | 2.47 | 2.59 |
| MP | -5.17 | -1.90 | 3.27 | 3.54 | 1.64 | 0.61 | -3.54 | 0.82 | 0.82 | 1.77 | 2.16 |
| UO_2_-MP | -6.53 | -3.30 | 3.23 | 4.92 | 1.62 | 0.62 | -4.92 | 0.81 | 0.81 | 2.46 | 3.05 |
| SCZ | -6.75 | -2.78 | 3.97 | 4.77 | 1.99 | 0.50 | -4.77 | 0.99 | 0.99 | 2.38 | 2.40 |
| UO_2_-SCZ | -6.62 | -3.55 | 3.07 | 5.09 | 1.54 | 0.65 | -5.09 | 0.77 | 0.77 | 2.54 | 3.31 |
