# Supplementary material for: Complexation of uranyl (UO2)2+ with bidentate ligands: XRD, spectroscopic, computational, and biological studies
Source: PLoS One. 2021 Aug 19;16(8):e0256186. doi: 10.1371/journal.pone.0256186 (PMC8376047; doi:10.1371/journal.pone.0256186)
Supplement: S2 Table — (DOCX) [file pone.0256186.s008.docx]

**S2 Table.** Thermal data for UO_2_(II) complexes.

| complex | step | Temp. range | Weight loss %  found(calc) | Assignments | Total mass loss/%  found(calc) | Final solid  state residue |
| --- | --- | --- | --- | --- | --- | --- |
| [UO_2_(CMZ)(ACO)_2_]. 2H_2_O | 1st | 140-173 | 5.9  (6) | 2H_2_O | 45.5  (46.4) | UO_2_ +  38.7  (38.6) |
|  | 2nd | 244-311 | 39.6  (40.4) | 2CNS+4C_2_H_2_ +2NO+N_2_ |  |  |
| [UO_2_(MP)(ACO)_2_] | One step | 182-240 | 47.4  (47) | 2CNS+4CN+4H_2_O | 47.4  (47) | UO_2_+C  51.86  (51.99) |
| [UO_2_(SCZ)(ACO)_2_] | One step | 176-245 | 55.5  (55.2) | C_14_H_15_O_8_N_4_SCl | 55.5  (56) | UO_2_  35.32  (34.95) |
