# Supplementary material for: Complexation of uranyl (UO2)2+ with bidentate ligands: XRD, spectroscopic, computational, and biological studies
Source: PLoS One. 2021 Aug 19;16(8):e0256186. doi: 10.1371/journal.pone.0256186 (PMC8376047; doi:10.1371/journal.pone.0256186)
Supplement: S3 Table — (DOCX) [file pone.0256186.s009.docx]

**S3 Table.** Kinetic data of metal complexes using Coats-Redfern and Horowitz and Metzger equation.

| complex | Temp. range C | Mothed | Parameter | | | | | Correlation  coefficient  (r) |
| --- | --- | --- | --- | --- | --- | --- | --- | --- |
|  |  |  | Ea  (kJmol^−1^) | A  (s^−1^) | -ΔS  (J mol^-1^ K^-1^) | ΔH  (kJmol^−1^) | ΔG  (kJ mol^−1^) |  |
| [UO_2_-CMZ(ACO)_2_]H_2_O | 140-173 | CR  HM  Average | 2.42×10^4^  7.78×10^4^  5.10×10^4^ | 1.48×10^4^  6.44×10^4^  3.96×10^4^ | 1.70×10^2^  1.58×10^2^  1.64×10^2^ | 1.95×10^4^  7.31×10^4^  4.63×10^4^ | 1.17×10^5^  1.63×10^5^  1.40×10^5^ | 0.9866  0.9990  0.9928 |
|  | 244-311 | CR  HM  Average | 1.80×10^4^  6.78×10^4^  4.29×10^4^ | 8.11×10^4^  6.88×10^3^  4.40×10^4^ | 1.56×10^2^  1.77×10^2^  1.67×10^2^ | 1.33×10^4^  6.31×10^4^  3.82×10^4^ | 1.02×10^5^  1.64×10^5^  1.33×10^5^ | 0.9615  0.9668  0.96415 |
| [UO_2_-MP(ACO)_2_] | 182-240 | CR  HM  Average | 2.82×10^4^  7.16×10^4^  4.99×10^4^ | 4.60×10^3^  2.79×10^5^  1.42×10^5^ | 1.79×10^2^  1.45×10^2^  1.62×10^2^ | 2.41×10^4^  6.76×10^4^  4.58×10^4^ | 1.11×10^5^  1.38×10^5^  1.25×10^5^ | 0.9819  0.9791  0.9805 |
| [UO_2_(SCZ)(ACO)_2_] | 176-245 | CR  HM  Average | 2.56×10^4^  6.93×10^4^  4.74×10^4^ | 8.25×10^3^  1.90×10^3^  5.08×10^3^ | 1.74×10^2^  1.48×10^2^  1.61×10^2^ | 2.16×10^4^  6.53×10^4^  4.34×10^4^ | 1.05×10^5^  1.37×10^5^  1.21×10^5^ | 0.9828  0.9805  0.9817 |
