# Supplementary material for: Complexation of uranyl (UO2)2+ with bidentate ligands: XRD, spectroscopic, computational, and biological studies
Source: PLoS One. 2021 Aug 19;16(8):e0256186. doi: 10.1371/journal.pone.0256186 (PMC8376047; doi:10.1371/journal.pone.0256186)
Supplement: S4 Table — (DOCX) [file pone.0256186.s010.docx]

**S4 Table.** Horowitz–Metzger (HM) and Coats–Redfern (CR) of metal complexes.

| CR method | HM method | Temp. range ºC | Complex |
| --- | --- | --- | --- |
|  |  | 140-173 | [UO_2_(CMZ)(ACO)_2_]. 2H_2_O |
|  |  | 244-311 |  |
|  |  | 182-240 | [UO_2_-MP(ACO)_2_] |
|  |  | 176-245 | [UO_2_(SCZ)(ACO)_2_] |
